# Enablers and barriers for scaling up non-communicable disease interventions across diverse global health contexts: a qualitative study using the Consolidated Framework for Implementation Research

**DOI:** 10.1136/bmjopen-2025-101292

**Published:** 2025-12-10

**Authors:** Zinzi Pardoel, Ilona Folkertsma, Anusha Ramani-Chander, Amanda G Thrift, Rohina Joshi, Isobel Bandurek, Josefien van Olmen, Abha Shrestha, Lal B Rawal, Edwin Wouters, Asri Maharani, Peter Delobelle, Hueiming Liu, Michaela Theilmann, Jacqui Webster, Sujarwoto Sujarwoto, Kamran Siddiqi, Ari Probandari, Vitri Widyaningsih, Jaime Miranda, Puhong Zhang, Lisa Stehr, Lisa R Hirschhorn, Jaap Koot, Manna Alma

**Affiliations:** 1 Department of Health Sciences, University Medical Center Groningen, University of Groningen, Groningen, Netherlands; 2 Department of Medicine, School of Clinical Sciences at Monash Health, Monash University, Clayton, Victoria, Australia; 3 Medicine, Monash University Stroke and Ageing Research Group, Clayton, Victoria, Australia; 4 School of Population Health, University of New South Wales (UNSW), Sydney, New South Wales, Australia; 5 Global Alliance for Chronic Diseases, London, UK; 6 Department of Primary and Interdisciplinary Care, University of Antwerp, Antwerp, Belgium; 7 Department of Community Medicine, Kathmandu University School of Medical Sciences, Dhulikhel, Nepal; 8 Dhulikhel Hospital, Dhulikhel, Nepal; 9 Western Sydney University, Sydney, New South Wales, Australia; 10 University of Antwerp, Antwerp, Belgium; 11 Nursing, Midwifery and Social Care, The University of Manchester, Manchester, UK; 12 University of Cape Town, Rondebosch, South Africa; 13 The George Institute for Global Health, Sydney, New South Wales, Australia; 14 Faculty of Medicine, Hillah, Iraq; 15 Heidelberg University, Heidelberg, Germany; 16 Public Administration, Brawijaya University, Malang, Indonesia; 17 University of York, York, UK; 18 Public Health, Universitas Sebelas Maret, Surakarta, Indonesia; 19 Faculty of Medicine Universitas Sebelas Maret, Surakarta, Indonesia; 20 The University of Sydney Faculty of Medicine and Health, Sydney, New South Wales, Australia; 21 The George Institute for Global Health UK, Oxford, UK; 22 Medical Social Sciences, Northwestern University Feinberg School of Medicine, Chicago, Illinois, USA; 23 Northwestern University Feinberg School of Medicine, Chicago, Illinois, USA

**Keywords:** Hypertension, Cardiovascular Disease, Diabetes Mellitus, Type 2, Chronic Disease, Implementation Science

## Abstract

**Objectives:**

To identify enablers and barriers for scaling up non-communicable disease (NCD) interventions across diverse global contexts and to map these factors to the WHO’s health system building blocks.

**Design:**

A multi-method qualitative study applying the Consolidated Framework for Implementation Research to analyse data from multiple projects nearing or completing scale-up.

**Setting:**

Global Alliance for Chronic Diseases-funded implementation research projects conducted across 18 low- and middle-income countries and high-income settings.

**Participants:**

Data was derived from documents (n=77) including peer-reviewed publications, policy briefs, and reports and interviews with stakeholders (n=18) (eg, principal investigators, medical professionals, public health workers).

**Interventions:**

Various context-specific interventions targeting sustainable scale-up of NCD (eg, diabetes, hypertension, cardiovascular disease) interventions at the community, primary care or policy levels.

**Primary and secondary outcome measures:**

The primary outcome was identifying contextual enablers and barriers to intervention scale-up. Secondary outcomes included exploring how these factors aligned with health system building blocks (eg, leadership/governance, healthcare workforce).

**Results:**

Twenty enablers (eg, intervention adaptability, strong stakeholder engagement, local empowerment) and 25 barriers (eg, resource limitations, intervention complexity, stakeholder burnout) were identified. Contextual alignment, supportive governance and capacity building were critical for sustainability, while cultural misalignment and socio-political instability frequently hampered scaling efforts.

**Conclusions:**

Tailoring interventions to local health systems, ensuring stakeholder co-ownership and incorporating strategies to mitigate stakeholder burn-out are essential to achieving sustainable, scalable NCD solutions. Future research should focus on integrating systematic cultural adaptation, sustainable financing and workforce capacity building into scale-up planning.

**STRENGTHS AND LIMITATIONS OF THIS STUDY**

- Multi-method approach: we combined document analysis with stakeholder interviews, enabling triangulation of data and a more comprehensive assessment of enablers and barriers.
- Application of the Consolidated Framework for Implementation Research: using a well-established framework helped systematically categorise multi-contextual implementation factors, enhancing transferability of the findings.
- Diverse project sample: the inclusion of 15 projects from multiple regions strengthens the global relevance of the results.
- Potential selection bias: projects that responded may have been more successful or motivated, thus underrepresenting barriers faced by less successful implementations.
- Limited stakeholder perspectives: most interviewees were investigators or health professionals; input from patients, community members and policymakers was limited.

## Introduction

 Non-communicable diseases (NCDs) such as cardiovascular disease, diabetes and certain cancers account for 74% of all deaths worldwide, with a disproportionate burden in low-income and middle-income countries (LMICs).^1^,^3^ Health service-level and contextual factors in LMICs and barriers faced by families with vulnerabilities or complex needs in high-income countries (HICs), such as ethnic minorities and low-income individuals, worsen inequalities in NCD prevention and treatment.^4^,^6^ Vulnerable families or those with complex needs face additional barriers, such as limited access to healthcare, nutritious food and safe housing, contributing to higher rates of NCDs and worse outcomes. Despite ongoing efforts,^7 8^ a significant gap in knowledge regarding the sustainable scale-up of NCD interventions in these contexts remains.^9^

Scaling up involves not just expanding the reach of successful programmes but also improving their effectiveness and institutionalising them to ensure long-term sustainability.^10^ Achieving sustainable scaled-up NCD interventions, particularly in LMICs, involves addressing complex barriers, such as limited healthcare infrastructure, workforce shortages and socioeconomic and cultural barriers, while strengthening local capacity and improving primary healthcare systems. These efforts align closely with the WHO’s six health systems building blocks,^11^ which provide a framework for strengthening health system performance. By integrating interventions into existing services, supporting a skilled workforce, enabling monitoring through health information systems, improving access to medicines, securing sustainable financing and fostering leadership, the building blocks guide scalable and sustainable solutions tailored to local contexts.^11^,^16^ 

Previously, Ramani-Chander *et al*^17^ analysed enablers and barriers to scaling up NCD interventions in LMICs and families with vulnerabilities in HICs, using systems thinking. Their research was conducted by examining projects funded by the Global Alliance for Chronic Diseases (GACDs), a global network that supports implementation research to address the prevention and treatment of NCDs in LMICs and vulnerable populations.^18 19^ The GACD-funded projects aimed to develop and scale up sustainable, community-based interventions targeting NCDs, leveraging diverse approaches adapted to local needs. They found that strong local partnerships and integration into existing systems supported scale-up, but limited infrastructure, inconsistent policy support and resource constraints posed major barriers. Political instability, leadership changes and fragmented health systems further disrupted implementation. Securing sustainable resources for medications and equipment remained a significant challenge. Key gaps included misalignment between national policies and local implementation, limited understanding of research’s role among policymakers and coordination challenges in multi-sectoral interventions. Ramani-Chander *et al*^17^ emphasised the need for context-specific frameworks to prioritise and plan scale-up research projects effectively. They also noted that implementation challenges were compounded by the limited guidance on balancing innovation with feasibility, misalignment between scalability and readiness assessments, and the absence of long-term sustainability strategies. Importantly, their study was conducted during the implementation phase of the GACD projects, leaving gaps in understanding the long-term outcomes of these interventions and their sustainability after project funding ended.

Building on insights and gaps identified in the preliminary research,^1720^,^22^ this follow-up study extends earlier findings on enablers and barriers to scaling up NCD interventions. The first study applied systems thinking to identify challenges during implementation, while the second focused on prioritising and planning for scale-up. Both contributed to the development of an extraction tool for systematically capturing factors influencing scale-up, including intervention characteristics, adaptation, stakeholder consultation and sustainability. In the present study, we apply this tool within the CFIR V.2.0 framework to examine projects that were nearing or completing scale-up and systematically map barriers and enablers to the WHO health system building blocks. In line with earlier scale-up research,^23^ we conceptualise scale-up in terms of expansion, coverage and institutionalisation. We recognise that expansion may overlap with implementation activities, but highlight that scale-up extends beyond initial delivery to embed interventions more broadly and sustainably within health systems. Although others have highlighted enablers and barriers to scaling up NCD interventions in LMICs, achieving sustainable and widespread implementation continues to pose significant challenges. By focusing on health system factors critical for scalability and sustainability, this study provides new insights using a framework into long-term strategies for embedding and expanding NCD-related initiatives across diverse contexts. Rather than focusing solely on research implementation or project management, we examined health system factors critical for scalability and sustainability. Our aim was to identify the enablers and barriers to scalability and explore their implications for the long-term sustainability of NCD interventions, offering valuable insights into effective and sustainable strategies for scaling up NCD-related initiatives in diverse contexts.

## Methods

### Study design

**Figure 1:**
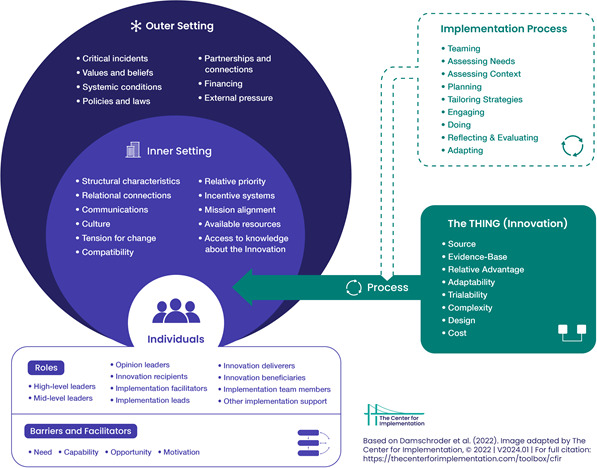
Consolidated framework for implementation research V.2.0 framework domains, description and constructs, adapted from Damschroder *et al*.^22^

A phased approach with multiple qualitative methods was used, combining document analysis, stakeholder interviews and data synthesis. We used the Consolidated Framework for Implementation Research (CFIR) V.2.0,^24^ which provides a structured, comprehensive method for examining the various enablers and barriers of implementing and scaling up health interventions,^24^ to guide data collection and analysis. This study was approved by the Monash University Human Research Ethics Committee (HREC number 23482). To ensure alignment with the peer-reviewed update, we used the original CFIR V.2.0 visual published by Damschroder *et al* (figure 1), which illustrates the five domains and corresponding constructs. This framework provided a structured lens for identifying and analysing barriers and enablers of scalability across projects. We applied CFIR V.2.0 as our primary framework because it provides a comprehensive structure for identifying barriers and enablers with a focus on scalability and sustainability. The WHO building blocks^11^ offer a systems-level perspective but are less detailed for implementation processes. To combine these strengths, we analysed data using CFIR V.2.0 and subsequently mapped the findings to the WHO building blocks.

### Sample and data collection

In 2019, the GACD scale-up call funded 27 projects, through eight funding agencies, investigating sustainable scale-up strategies for NCD interventions.^19^ All 27 projects targeted NCD treatment, prevention and public health, using community-based interventions, primary care and policy collaboration to improve outcomes. The strategies comprised enhancing awareness, screening and management while adapting to local needs. This common goal enabled diverse approaches to be tested and scaled across various contexts and settings.^21 22^ In this study, we included only projects that were considered to be ‘nearing or completing scale-up’. Of the original 27 GACD-funded implementation research projects, 6 were excluded because they had not yet reached this stage. Projects were considered to be ‘nearing or completing scale-up’ if they had reached key expansion milestones beyond initial pilot implementation. These milestones included extending interventions to multiple districts or provinces, formal adoption within regional or national health systems, or integration into national policy frameworks. Because of contextual differences, no single pre-specified target for scale-up was applied across projects; rather, the definition of scale-up was based on progress toward embedding interventions beyond pilot phases within health systems. This follow-up study included the 27 GACD studies included in the preliminary research,^17 20^ with data collected from March 2024 to July 2024. The scale-up working group, consisting of researchers from the NCD implementation and scaling up studies,^1720^,^22^ GACD partners and researchers from the University Medical Center Groningen, the Netherlands, led this follow-up study and contacted all relevant project stakeholders from the 27 projects. Six projects were excluded due to insufficient implementation, no scaling-up efforts or unavailable documentation. After four contact attempts, no responses were received from another six projects. Therefore, the study included 15 projects nearing or completing scaling up. Initial contacts were made with project representatives to gather relevant documents, for example, midterm and final reports, peer-reviewed publications and policy briefs. After representatives provided written informed consent, documents were shared via email. Once the document analyses were completed, interviews were conducted.

Data collection involved a combination of document review and semi-structured interviews for each project. Interviews were conducted with project representatives to complement the documentary data, focusing on information that was underreported, unclear or missing in the documents (eg, stakeholder perspectives, contextual adaptations, sustainability planning). This linkage ensured that the interview and document data were integrated and project-specific.

Table 1 provides a descriptive overview of the projects included in the dataset (eg, WHO region, intervention type, number of documents). These characteristics are presented here to contextualise the dataset. The projects represented 18 countries across five WHO regions: African Region (AFR), South-East Asian Region (SEAR), Region of the Americas, European Region (EUR) and Western Pacific Region (WPR). The project duration varied from three to six years and comprised diverse focuses, target populations and diseases.

**Table 1:** Overview of characteristics of the study sample

	WHO regions*					Multi (M) or single (S) country	Duration				Primary focus					Target group			Disease			
	South-East Asian Region	African Region	European Region	Western Pacific Region	Region of the Americas		3 years	4 years	5 years	6 years	All	Policy	Primary care	Community	System	General population	Diagnosed with disease	At risk of NCD(s)	Hypertension	Cardiovascular disease	Diabetes	Multiple NCDs
Study 1	✔		✔			M		✔			✔					✔	✔	✔	✔		✔	
Study 2	✔		✔			M		✔			✔					✔		✔	✔		✔	
Study 3		✔				S			✔				✔			✔		✔	✔		✔	
Study 4					✔	S	✔					✔	✔		✔			✔	✔			
Study 5					✔	S		✔				✔			✔	✔			✔		✔	
Study 6	✔					S		✔						✔			✔				✔	
Study 7				✔		S			✔					✔		✔			✔	✔		
Study 8		✔				S	✔					✔	✔	✔			✔					✔
Study 9	✔					S			✔					✔				✔	✔			
Study 10					✔	S				✔			✔				✔		✔		✔	
Study 11					✔	M			✔			✔				✔						✔
Study 12	✔					S		✔					✔					✔	✔			
Study 13		✔				S	✔						✔				✔				✔	
Study 14		✔				S	✔						✔	✔		✔						✔
Study 15		✔				S				✔		✔						✔	✔			

* WHO regions presented in this table reflect the geographic coverage of the interventions at the time of data collection. In some cases, projects expanded from their original site into multiple countries or regions, while others remained within the initial WHO region where they were piloted.

NCDs, Non-communicable diseases.

### Document analysis

A total of 77 documents were shared, including white papers (n=2), reports (n=23), policy briefs (n=5), publications (n=39), infographics (n=2) and others (n=6), for example, research protocols and conference papers. Between one and 14 documents were shared per project, averaging five per project. Document analysis was conducted using the extraction tool developed during the initial studies.^17 20^ The extraction tool incorporated seven key categories of enablers and challenges of sustainable scaling-up NCD interventions found in the preliminary studies, that is, intervention, innovation, local context, environment, local adaptation, stakeholder consultation and sustainability. These key elements were integrated into the CFIR framework per project to get a structured overview of how they influence scaling-up of interventions (online supplemental file 1).

### Stakeholder interviews

Stakeholders from 14 out of 15 projects were interviewed. For one project, no stakeholder was interviewed due to technical issues on three attempts, and only the documents were included. To capture a multi-stakeholder perspective, up to three stakeholders per project were included. Stakeholders were identified through a contact person known by the GACD or from preliminary studies. They were eligible if they had been involved to a certain extent in the project, ensuring they had sufficient knowledge of its development, implementation and scaling-up. Stakeholders were approached via email, either directly by the researchers or through the project contact person. In total, 18 stakeholders were interviewed, including academics, investigators or researchers (n=15), medical professionals (n=2) and public health workers (n=1). Interview guides from the initial study were modified to better suit the follow-up stage (eg, adding questions that explored the sustainability of outcomes after the end of a project). The interviews focused on underreported or unclear information from documentation related to the seven key categories described earlier (online supplemental file 2). Researchers ZP (female, PhD, social scientist) and IF (female, MSc, social scientist), who had no prior relationships with any of the interviewed stakeholders before the study, conducted the interviews, each lasting 45 to 60 min. All interviews were held online, recorded and transcribed using the Teams (Microsoft 360) application, with the participants’ permission. Each transcript was checked for accuracy and completeness by ZP or IF.

### Patient and public involvement

None.

### Data integration and analysis

Findings from the document analysis and interviews were integrated into an Excel sheet per project. Framework analysis,^25^ that is, a systematic method that enables structured comparison and detailed analysis across datasets, was used to organise and interpret data based on predefined or emerging themes, structured around seven key categories linked to CFIR domains (online supplemental file 1). ZP and IF manually coded the data in Excel to identify recurring themes, barriers, successes and factors related to scaling up and sustainability. For reporting purposes, we applied plain-language labels to the identified barriers and enablers to ensure accessibility for global health practitioners and policymakers less familiar with CFIR terminology. To enhance transparency and alignment with the original framework, (online supplemental file 3) provides the corresponding CFIR V.2.0 construct names and definitions for each barrier and enabler. To address our secondary objective, we also systematically mapped the identified enablers and barriers from CFIR domains to the WHO’s six health system building blocks: service delivery, health workforce, health information systems, access to essential medicines and technologies, financing and leadership/governance. This deductive mapping approach was conducted in Excel, whereby each CFIR-coded barrier or enabler was assigned to one or more relevant WHO building blocks. Where enablers or barriers aligned with more than one building block, they were noted as overlapping to highlight cross-cutting relevance (online supplemental file 4). To ensure robustness and credibility of the findings, results were cross-validated and triangulated by ZP and IF. Additionally, peer debriefing sessions were held with MA and JK to critically reflect on the coding process and interpretations. Interim analyses were also discussed with the entire research group or steering committee, providing broader validation and further insights into the findings. Other authors reviewed the interpretation of the findings.

## Results

**Figure 2:**
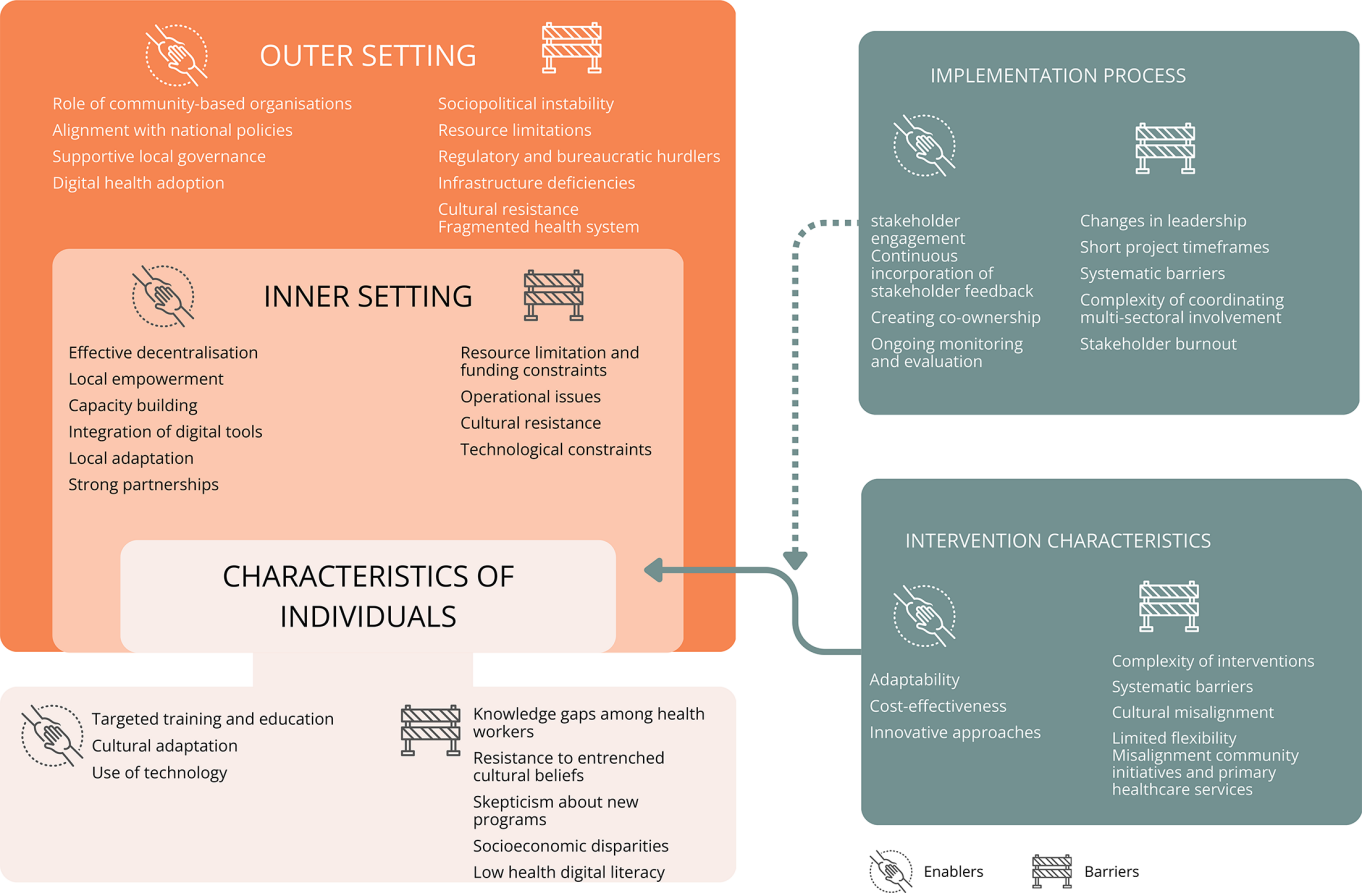
Overview of the enablers and barriers by the consolidated framework for the implementation research domain.^22^

In total, we identified 20 enablers and 25 barriers in scaling up NCD-related interventions, presented by CFIR domain (figure 2, online supplemental file 5).

### Intervention characteristics

Within the *Intervention characteristics* domain, we identified several enablers and barriers across various countries. *Adaptability*, referring to how well interventions can be modified to fit local contexts, healthcare infrastructure and cultural needs, emerged as a crucial enabler. Stakeholders emphasised that interventions tailored to local settings proved more sustainable, were more likely to be accepted by local stakeholders and ensured easier integration into existing healthcare provision. Adapting interventions to local contexts enhanced acceptability and participation among community members (illustrated by Quote 1). Moreover, *cost-effectiveness* facilitated scalability by reducing resource strain, as seen in multiple projects. For example, in the region of the Americas (AMR), the integration of an innovative programme reduced costs and complexity, making it feasible for long-term sustained scale-up. *Innovation*, that is, the introduction of new approaches in the projects for managing NCDs or integrating services, was another enabler of scalability, as novel methods supported adaptation across contexts and long-term embedding within health systems. Examples included the use of digital health tools to extend care delivery, community-based delivery models to increase reach, and cross-sectoral collaborations to integrate NCD care into existing health and social services. Such innovations enhanced adaptability and facilitated scalability across diverse contexts. However, stakeholders emphasised the need to support these approaches with evidence to gain broad acceptance.

We also made sure that women who are older will be able to speak in their own language because they usually don’t speak other languages. And once we were in the field, we didn’t know exactly whether it was going to be important, but once we were in the field, we found that it was really key for older women to participate and feel comfortable talking in their own language, for instance. (Quote 1, Academic)

In contrast, the *complexity of interventions* was a significant barrier to scalability and sustainability, whereby resource-intensive interventions requiring extensive coordination or training created challenges for expansion into multiple settings and for maintaining fidelity over time. Moreover, *systematic barriers* such as resource constraints were frequently noted. Stakeholders suggested that better assessments of local resources before intervention rollout would be essential to mitigate such barriers. Further, *cultural misalignment* also posed a significant barrier, where interventions that did not align with local cultural norms, values or healthcare practices faced resistance from both healthcare workers and community members. For example, in a project in AMR, aspects of the intervention that clashed with local health practices led to push back from both healthcare workers and patients. This resistance stemmed from a perceived disconnect between the intervention design and the community’s lived experiences or traditional practices. Disconnects between intervention design and communities’ lived experiences emerged as barriers not only during initial implementation but also as projects expanded into new settings. Cultural sensitivity is an ongoing need, essential from the outset and throughout sustainability to ensure acceptance and engagement. Stakeholders emphasised the need for culturally sensitive interventions to ensure acceptance, sustainability and scalability. While aligning with cultural contexts is vital, they noted that achieving broader health goals may sometimes necessitate gradual adjustments to local practices, reinforced through community engagement and ongoing collaboration to sustain scale-up. They highlighted that such changes can be achieved through community engagement, trust-building and gradual collaboration with local leaders and healthcare workers to ensure acceptance and sustainability. An additional barrier identified was the *misalignment between community initiatives and primary healthcare services*, which posed a barrier to effective service delivery (illustrated by Quote 2). Stakeholders expressed that community-led initiatives that did not align with the protocols and services offered by PHC facilities led to confusion and inefficiencies in patient care. Finally, *limited flexibility* was another barrier, whereby interventions that lacked adaptability to different contexts had significant impediments in gaining traction.

There is not always harmonious relationships between community-based and facility-based health services. Community health workers, who operate in these communities and are not always highly regarded by professional health practitioners like nurses, often experience a ‘down referral’ system from facilities to communities rather than the other way around. (Quote 2, academic)

### Outer setting

Within the domain of the *Outer setting*, enablers included the crucial *role of community-based organisations*. These organisations mobilised hard-to-reach populations and integrated interventions into local social structures. For instance, a community-based organisation in the SEAR was essential in connecting older populations with health programmes, ensuring that interventions were embedded in social systems that people trust and engage with regularly. Quote 3 further illustrates this enabler, highlighting how community health workers, when given a voice and space to participate actively, strengthened ownership and made interventions work effectively. Another enabler found was the *alignment with national policies*. In a project in the AFR, early engagement with policymakers through working group meetings was a consistent enabler of effective programme delivery, while in another project in AFR, multi-sectoral stakeholder meetings were vital for aligning health programmes with standardised guidelines. Moreover, *supportive local governance* was identified as a strong enabler. In a project in AMR, the engagement of a committed health secretary helped ensure the scalability and sustainability of health initiatives, and in a project in SEAR, local government cooperation facilitated programme integration into existing health structures for long-term expansion. During the COVID-19 pandemic, *digital health adoption* accelerated. In projects in the AFR, AMR and WPR, digital platforms were critical for maintaining healthcare delivery during lockdowns. In a project in SEAR, the adoption of mobile health applications bridged communication gaps between health workers and rural communities, enabling continued service delivery amidst the pandemic.

The fact that community health workers will come in groups and sit actively in meetings… they just they are… what they said in their own words, is that they were given a voice. This was something that really made the whole thing work. (Quote 3, medical professional)

In contrast, barriers were frequently mentioned across several projects, particularly *socio-political instability*. In a project in SEAR, a military coup caused severe delays in healthcare interventions, while in a project in AMR, civil unrest similarly disrupted timelines. In a project in AFR, political unrest was mentioned as a barrier to securing consistent government support for long-term health interventions. Another barrier found was *resource limitations*, briefly discussed in the *Intervention characteristics*, in which the COVID-19 pandemic exacerbated this barrier with shifting priorities. In these projects, resources were redirected towards the pandemic response, causing delays in other health initiatives. Furthermore, *regulatory barriers* and *bureaucratic hurdles* were found. In a project in AFR, weak food labelling regulations and bureaucratic delays were mentioned as major obstacles. In a project in SEAR, navigating local regulatory frameworks posed a barrier, particularly when attempting to align with global health guidelines. In a project in the EUR, bureaucratic delays, with slow regulatory approvals, hindered the scaling up of digital health interventions. Other barriers mentioned were *infrastructural deficiencies* and *fragmented health systems*, in which the lack of integration among various levels of healthcare often results in poor patient follow-up and continuity of care and rural populations facing barriers to accessing health services (illustrated by Quote 4). Lastly, *cultural resistance* discussed previously was frequently mentioned, especially in rural areas.

The availability of NCD drugs in public hospitals is low, at 32%, but it gets even worse in primary care facilities where it is 25%. Frequent stock outs and high mark-ups, sometimes up to 500%, remain major barriers to equitable access. (Quote 4, Policy brief)

### Inner setting

In the *Inner setting* domain, we identified *effective decentralisation* and *local empowerment* as significant enablers, enhancing local decision-making and aligning interventions with specific community needs (illustrated by Quote 5). These enablers were noted in projects across SEAR, where health services operated at the provincial and district levels, allowing for tailored community interventions. Some stakeholders emphasised that local empowerment not only improved responsiveness but also fostered greater community engagement and ownership of health initiatives. However, it should be noted that decentralisation, while facilitating local empowerment, may pose challenges for scaling up interventions, as centralised and vertical programmes are often more efficient in achieving broader, uniform implementation. In the inner setting, *capacity building* and the *integration of digital tools* further supported scalability and sustainability by fostering a skilled workforce and facilitating ongoing adaptation. Stakeholders recognised training as essential for equipping health workers with the necessary skills to address NCDs effectively. In projects in SEAR, *local adaptation* increased their relevance and effectiveness at the level of the internal organisational environment, which was also mentioned in the *Intervention characteristics domain*. Additionally, *strong partnerships with local stakeholders* reinforced the stability and long-term feasibility of these interventions. Document analysis revealed that successful collaborations often led to shared resources and improved coordination among health services, which stakeholders highlighted as critical for sustaining health programmes.

Local decision-making has transformed our approach; with decentralisation, we can tailor health interventions to meet the specific needs of our communities. This has empowered us to engage more effectively with local stakeholders. (Quote 5, academic)

Barriers included *resource limitations* and *funding constraints*, as previously highlighted in the *Intervention Characteristics domain*. *Operational issues* affected five projects in SEAR, highlighting that healthcare workers often felt overwhelmed and underprepared to tackle the complexities of NCD management due to insufficient training. Turnover of staff further exacerbated these challenges, as continuity of care was disrupted and institutional knowledge was lost (illustrated by Quote 6). Lastly, *cultural resistance* discussed previously, and *(digital) technological constraints* also presented significant barriers, impacting projects across SEAR and AFR.

I mean like you can train five people in a facility and 6 months later none of them are available. Somebody has resigned and gone off somewhere, you know, etcetera. It’s amazing how quickly you could end up with nobody still available to run the session, so there’s a huge need to train the right people, train enough people and continue offering training. (Quote 6, academic)

### Individual characteristics

In the domain of *Individual characteristics*, enablers included *targeted training and education*, significantly enhancing healthcare workers’ knowledge and confidence. This enabler was also highlighted in the *Inner Setting* domain, where capacity building through training played a key role in sustainability. We also found that, as previously noted, adaptability and cultural sensitivity enhanced the *acceptance *of interventions (illustrated by Quote 7). Acceptance by the target population can lead to greater trust, reduced resistance and increased engagement in the intervention, which are essential for scaling up and sustainability. This echoes the importance of adaptability discussed under *Intervention characteristics*, where interventions tailored to local contexts were better accepted and more sustainable. Another enabler was the *use of technology*, such as mobile health apps and decision support systems, which strengthened both healthcare providers’ and patients’ self-efficacy, as highlighted under the *Outer setting and Inner setting* domains.

Traditional diets, you know, anything traditional, we should know it because going out to the community, that’s the first thing we need to learn is how the different choices or the current practices are in each of the communities. It’s really about understanding the community before we go into their own homes, because you know there is specially like some part from traditional, it’s also their belief something that we needed to also understand. (Quote 7, academic)

Several barriers were revealed, such as *knowledge gaps among healthcare workers*, a barrier also discussed in the *Inner setting* domain, where inadequate training limited the ability to manage NCDs effectively (illustrated by Quote 8). Additionally, *cultural resistance*, previously mentioned in both the *Intervention characteristics* and *Outer setting* domains, hindered the acceptance of interventions. Within *individual characteristics,* cultural resistance stemmed from personal scepticism. By contrast, in *Intervention characteristics*, resistance arose from practices misaligned with local traditions, while in the *Outer setting*, resistance was tied to broader socio-political norms, such as gender roles. Moreover, *scepticism about new health programmes* hindered progress in three projects across SEAR, AFR and AMR. *Socioeconomic disparities*, particularly pronounced in LMICs, complicated individuals’ abilities to engage with interventions, echoing resource barriers highlighted earlier. A stakeholder from a project in AMR indicated that economic factors, particularly household-level and individual-level financial constraints such as limited income, restricted access to healthy foods and medicines, complicate the promotion of healthier lifestyles. These constraints not only affected individual participation but also posed broader challenges to the scalability and sustainability of interventions in resource-limited settings. Lastly, *low digital literacy* was identified as a barrier in projects in SEAR and AFR. This was particularly pronounced in LMICs, where it was a persistent barrier that mirrored the difficulties with digital adoption mentioned in the *Outer setting*.

When we don’t understand the guidelines or best practices, it undermines our efforts to engage patients and promote healthy behaviours. (Quote 8, academic)

### Implementation process

In the domain of the *Implementation process*, enablers included strong *stakeholder engagement*, with stakeholders underscoring the value of early and ongoing involvement of local healthcare professionals, government bodies and community leaders (illustrated by Quote 9). Related to this, we found *continuous incorporation of stakeholder feedback* throughout the interventions’ planning, execution and later stages of scale-up was an enabler ensuring long-term sustainability and adaptability by allowing interventions to be adapted in real time to meet local needs and barriers. This mirrors the results under *Intervention characteristics*, where strong, early and ongoing stakeholder involvement was identified as being crucial for the success of scaling up and sustainability. Another enabler was the creation of *co-ownership* among local stakeholders. Actively involving them in decision-making and implementation helped build a sense of responsibility and long-term commitment, similar to the enabler of local empowerment highlighted in the *Inner setting* domain. *Ongoing monitoring* and *evaluation* were also highlighted as essential for the projects’ success, where stakeholders acknowledged the importance of feedback loops in refining intervention strategies, allowing for timely adjustments and improvements.

Which means that it will affect their economy of the household, so we need to work quite a lot with the community on designing and implementing this intervention. Because we are scaling up, we need to develop and cocreate with the community and stakeholders. (Quote 9, academic)

However, barriers persisted, such as *frequent changes in leadership* in government and project management, that can disrupt policy continuity and stakeholder commitment, delaying the scale-up process. Moreover, *short project timeframes* were identified as a barrier, limiting the ability to fully evaluate long-term impacts. Additionally, we found *systemic barriers* such as supply chain issues, lack of trained personnel and difficulties integrating new interventions into existing health systems (illustrated by Quote 10). These barriers were also seen under the *Inner setting* domain, where resource limitations and inadequate training were major obstacles. Another barrier was the *complexity of coordinating multi-sectoral involvement*, where managing diverse stakeholders from government agencies, industries and health organisations posed significant barriers, especially in the absence of existing structures for multisectoral collaboration. Finally, *stakeholder burnout* emerged as a significant barrier, as stakeholders felt overwhelmed by the demands of multiple initiatives, leading to reduced engagement.

An important aspect is our support decision systems are not integrated to the official medical recordings, so the professional needs to fill up our system and then to fill up this information is in the medical recording. So, it’s a big issue and we need to figure out a solution to. (Quote 10, medical professional)

### Mapping to WHO health system building blocks

**Figure 3:**
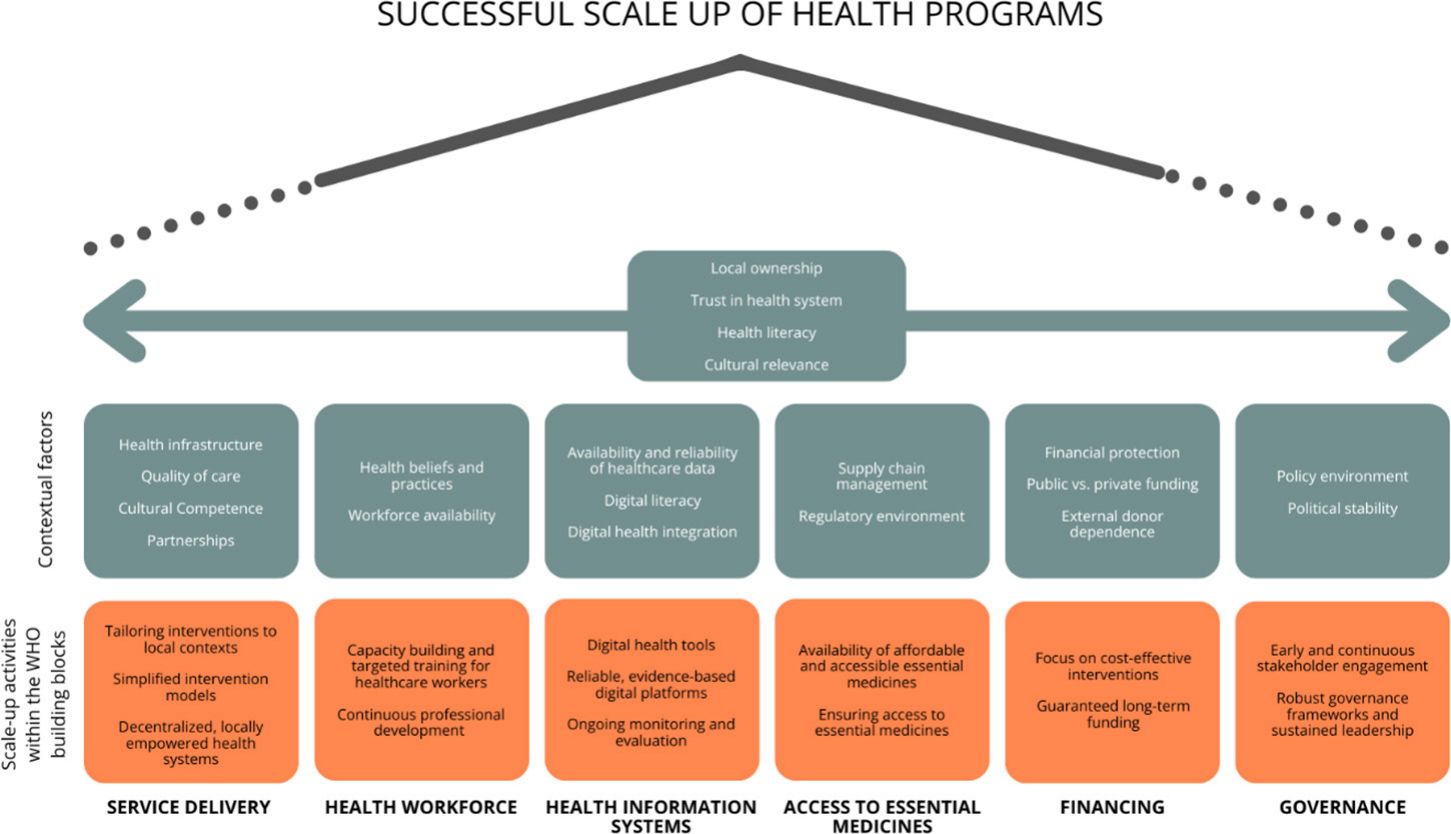
Key enablers for scaling up sustainable health programmes: alignment with the WHO’s six building blocks of health systems.^11^

The mapping exercise demonstrated a strong alignment between CFIR-identified barriers/enablers and the WHO building blocks (figure 3; Online supplemental file 4).

Service delivery was most influenced by factors such as adaptability and stakeholder engagement, each of which facilitated integration of interventions into existing services, while greater complexity of interventions diminished the capacity for their delivery. Health workforce mapping highlighted capacity building and targeted training as critical for sustainability, contrasted by knowledge gaps and staff turnover, which undermined continuity of care. Moreover, health information systems benefitted from integration of digital tools that supported care during the COVID-19 pandemic, but faced barriers including low digital literacy and infrastructural challenges. Further, medicines and technologies were linked to resource limitations and regulatory barriers, especially in contexts where weak systems delayed scale-up or diverted supplies. Financing overlapped with intervention characteristics, where cost-effectiveness supported sustainability, but funding constraints and short project timeframes limited scalability. Lastly, leadership and governance aligned strongly with policy alignment and supportive local governance, which facilitated long-term embedding of programmes. However, socio-political instability and stakeholder burnout reflected fragility in sustaining momentum. Several cross-cutting factors, particularly adaptability, stakeholder engagement and resource limitations, spanned multiple building blocks, underlining their systemic influence on scale-up efforts.

## Discussion

Our study demonstrates the complex dynamics involved in sustainable scaling up NCD-related interventions. Key enablers identified across the CFIR domains include adaptability, local empowerment and innovative approaches, all of which are crucial for enhancing the sustainability and effectiveness of these interventions. Barriers such as resource constraints, systemic barriers and cultural misalignment hinder successful scale-ups. Addressing these barriers requires effective stakeholder engagement and context-specific strategies. While these factors have been acknowledged in prior research, this study provides novel insights into how these enablers operate in practice, particularly in low-resource settings. For example, our findings highlight the importance of adaptability and the ongoing contextualisation of interventions to address cultural misalignment, a nuance not fully explored in earlier studies. While a few projects included participatory elements that resembled co-design, the dominant pattern was the adaptation of interventions to local contexts as they scaled. Additionally, the study sheds light on innovative approaches such as leveraging digital health tools to sustain interventions during disruptions, like those caused by the COVID-19 pandemic. These findings contribute to a deeper understanding of how these enablers can be practically applied to overcome specific barriers and achieve scalable, context-sensitive solutions.

Our findings not only highlight barriers and enablers across CFIR domains but also demonstrate how these factors align with the WHO’s six building blocks of health systems. As illustrated in figure 3 and detailed in online supplemental file 4, scalability was influenced by enablers such as adaptability, stakeholder engagement and cost-effectiveness, while barriers such as complexity, resource limitations and socio-political instability spanned multiple system components. This mapping provides an integrated perspective that connects implementation science with global health system frameworks, strengthening the applicability of our results for policy and practice. While our mapping highlighted enablers and barriers across the WHO building blocks, some contextual dimensions warrant further elaboration. For example, factors such as adaptability and acceptability may reflect broader aspects of quality of care, though these were often described implicitly rather than explicitly. Similarly, although financing emerged as a barrier, our analysis did not differentiate between public versus private financing sources or the role of external donor dependence, which may have significant implications for sustainability. Moreover, beyond health literacy at the individual level, the importance of health system literacy, that is, stakeholders’ ability to navigate governance, financing and delivery structures, was not fully captured. Finally, while stakeholder engagement and cultural alignment point to issues of trust, this theme was not explicitly developed in the data and remains a critical area for future research.

While healthcare systems were central to the sustainability of interventions, our findings also pointed to the importance of broader systems, including social and community structures, education systems, and policy/governance mechanisms. These intersecting systems played a critical role in enabling scale-up and sustainability, for example by mobilising communities, embedding interventions in trusted local networks and aligning health initiatives with broader social development priorities.

One significant finding was the critical importance of tailoring interventions to local contexts. Adapting interventions to existing healthcare infrastructures and cultural needs emerged as a pivotal enabler of both accessibility and sustainability, especially in resource-constrained environments. These findings resonate with the findings of Murray *et al*^26^ who emphasised that contextually relevant interventions improve outcomes. However, while flexibility and adaptation are essential for scaling up interventions, they must be balanced by preserving the evidence-based core of the interventions to ensure efficacy. Careful adaptation models, such as those discussed in the National Academies report,^27^ provide frameworks for modifying interventions to align with local needs while maintaining their theoretical and empirical integrity.

Intervention complexity was identified as a barrier, particularly in LMICs, where decentralised, locally empowered systems are essential for community-responsive care. This finding aligns with Peters *et al*^28^ and Gilson *et al*^29^ who stressed the importance of local flexibility in overcoming systemic barriers. While the participants in this study did not report this explicitly, the literature highlights an inherent tension: simplifying an intervention to enhance scalability may inadvertently dilute its evidence-based core. Models of cultural adaptation, such as Bernal and Sáez-Santiago’s ecological validity framework,^30^ provide a way to safeguard core components while addressing local sociocultural dynamics.

Stakeholder engagement was essential for fostering local ownership and ensuring the sustainability of interventions. For example, in one project, early collaboration with community leaders helped integrate dietary interventions into public health programmes, improving uptake and acceptability. However, challenges such as stakeholder burnout and political instability highlight the fragility of these efforts. Building on findings from Greenhalgh *et al*^31^ our findings support the introduction of stakeholder burnout as a critical barrier.

Overall, sustainable health interventions are shaped by the interconnected nature of the WHO’s six health systems’ building blocks. Stakeholder burnout and the interplay between financing and system components, while recognised in prior research, were strongly reinforced by our findings, particularly in LMIC contexts where these challenges were compounded by weak infrastructure and political instability. Future projects should incorporate systematic approaches to cultural adaptation, such as those proposed in Bernal *et al*’s framework,^30^ to ensure interventions remain both culturally appropriate and evidence-based. Scaling-up initiatives should embed a systems-thinking approach into their design and implementation from the outset, recognising that enablers and barriers are interdependent. For example, adaptability was closely tied to financing structures, stakeholder engagement influenced both governance and service delivery, and digital innovations depended on workforce capacity and infrastructure. Addressing these dynamics in a coordinated way is essential to strengthen long-term sustainability.

Building on the initial studies by Ramani-Chander *et al*^1720^,^22^ this research strengthens earlier findings on the role of intervention characteristics in scaling up NCD interventions, focusing on the longer-term impacts of COVID-19 and capturing required adaptations while providing deeper insights into enablers and barriers in low-resource settings. The initial investigators^1720^,^22^ highlighted the importance of tailoring interventions to local contexts, showing that adaptability enhances both sustainability and stakeholder acceptance. This follow-up study supports these conclusions, in that interventions adapted to local healthcare systems and cultural norms are more likely to succeed across diverse global settings. Likewise, stakeholder engagement continues to be crucial for fostering ownership and ensuring the long-term success of interventions. This study further contributes deeper insights into barriers such as intervention complexity and systemic barriers. While the initial research provided evidence that resource limitations and coordination issues were significant obstacles to scaling up interventions,^17^ findings from this follow-up study provide greater emphasis on the importance of adopting culturally sensitive ways to influence or modify local practices to avoid resistance from healthcare providers and patients. Moreover, we have introduced new insights into stakeholder burnout, an issue not fully addressed in the initial research. By identifying this gap, we recommend that future scale-up efforts should incorporate strategies to mitigate stakeholder burn-out and maintain engagement throughout the implementation process.

### Strengths and limitations

A strength of this study is applying the multi-dimensional CFIR. By focusing on populations in LMICs and families with vulnerabilities in HICs, the study provides valuable insights into how context-specific factors influence intervention success. This is crucial for developing targeted interventions and policies that address the unique needs of diverse populations, enhancing the sustainability and impact of NCD interventions globally. Triangulation of data collection methods enhanced the robustness and credibility of the findings in this study. By integrating seven key categories into the CFIR framework, document analysis provided a structured overview of the factors influencing the implementation and scaling of NCD interventions. Complementing this, stakeholder interviews addressed underreported information, ensuring diverse perspectives were captured. The integration of both methods allowed for the identification of recurring themes and barriers, while cross-validation by researchers strengthened the reliability of our conclusions, underscoring the value of using multiple data sources for a comprehensive understanding of complex implementation processes. Another strength of this study is the capturing of the complexity of scaling up NCD interventions, by identifying factors that span across multiple CFIR domains. This overlap provides a holistic view of the interconnected enablers and barriers influencing scalability and sustainability and provides a multi-domain perspective that enables a more nuanced understanding that can inform tailored, context-sensitive strategies, enhancing the study’s relevance across diverse settings. For example, adaptability was identified as an enabler across Intervention characteristics**,** Inner setting and Outer setting, highlighting its central role in facilitating local acceptance and integration of interventions. The cross-domain factors may be more relevant for scalability as they reflect the broader, systemic challenges that influence multiple aspects of implementation. These complexities require sophisticated analytical approaches to fully understand their roles and should be interpreted with care to ensure the nuanced relationships between domains are accurately represented.

A limitation is the lack of input from key stakeholders in the field, such as community members, nurses and policymakers. This limitation likely stemmed from the recruitment process, which primarily relied on invitations sent through the principal investigators. As a result, individuals who may have provided valuable insights into the implementation and sustainability of interventions were not adequately represented. Further, there may have been a bias, as principal investigators of more successful projects might have been more willing to share reports or agree to interviews, potentially leading to easier identified enablers and underrepresentation of barriers. This could limit insights into challenges faced by less successful or stalled projects, which often provide critical lessons. Lastly, given that most stakeholders participating in this study were people involved in the scaling-up projects, we could not capture ‘the outsider’s view’ or those affected by the interventions, such as clients or community members, of these scaling-up efforts. The absence of input from these critical voices may have limited the understanding of the real-world barriers and successes associated with the interventions. It also constrained our ability to fully evaluate CFIR constructs at the individual level, as perspectives from key actors were missing. Nevertheless, by focusing on projects nearing or completing scale-up, integrating our extraction tool with CFIR V.2.0 and mapping results to the WHO building blocks, we were still able to generate novel insights into systemic enablers and barriers influencing scalability and sustainability.

### Implications

This study provides insights that health planners can apply to scale up NCD interventions globally. A key takeaway message is that scaling efforts without considering the context of the broader health systems risks compromising sustainability. All scale-up initiatives should be approached with a health systems perspective to ensure long-term success and continued support. Integrating interventions into existing healthcare structures, strengthening local workforce capacity and addressing infrastructure gaps are essential for scalability. Aligning interventions with health system priorities such as service delivery, financing and governance is essential for scale-up, but not sufficient on its own. Even well-aligned programmes may encounter barriers beyond the control of implementers, including political instability, resource limitations or fragmented systems, which can ultimately hinder their impact. Policymakers and practitioners should therefore prioritise health system strengthening as a foundation for scaling up NCD interventions, while recognising that external factors may still pose challenges. In practice, early and sustained engagement of local stakeholders, including healthcare providers and community members, fosters ownership and supports long-term success. Strategies should also prevent stakeholder burnout, maintain engagement throughout the intervention lifecycle and simplify intervention models to reduce complexity, particularly in decentralised systems. Finally, addressing financial constraints through sustainable financing mechanisms and collaboration between policymakers and implementation teams is critical for ensuring continuity and expansion.

This study provides critical evidence of the necessity for research that delves deeper into context-specific strategies for adapting interventions to local healthcare systems and cultural contexts. Future research should focus on methodologies for systematically assessing local contexts and developing adaptable intervention frameworks that enhance community acceptance and effectiveness. Additionally, the complexity of interventions emerged as a significant barrier; thus, research should explore how to simplify intervention models without compromising their effectiveness, especially in resource-limited settings with fragmented healthcare systems. Furthermore, understanding workforce capacity is vital. Research should be designed to assess effective strategies for addressing workforce shortages and implementing sustainable professional development programmes, task-shifting and task-sharing opportunities, examining the impact of targeted training initiatives on health outcomes and intervention sustainability. Lastly, the development of sustainable financing models that support the interconnected components of health systems, such as service delivery, workforce capacity and governance, is critical. Longitudinal studies could offer insights into how various financing strategies influence the effectiveness and scalability of health interventions over time.

Our study was designed as a qualitative exploration of scale-up processes rather than an evaluation of intervention outcomes. While some included projects have quantitative effectiveness data reported elsewhere,^32^,^38^ we did not systematically assess effectiveness, and it remains unclear whether all projects maintained their intended benefits during scale-up. Therefore, our findings should be interpreted as insights into scale-up processes rather than assessments of intervention impact.

## Conclusion

This study advances implementation science by offering insights into the enablers and barriers of scaling up NCD interventions across diverse contexts. Novel findings, such as the role of stakeholder burnout and the interplay between intervention adaptability, digital health integration and local empowerment, highlight the complexity of achieving sustainable scalability. Addressing these knowledge gaps, including the need for context-sensitive approaches and alignment with national health policies, emphasises the importance of tailoring interventions to local systems and cultural norms. The methodological rigour of integrating document analysis and stakeholder interviews provides a robust foundation for understanding these dynamics. To ensure long-term success, future scaling-up efforts must prioritise capacity building, sustainable financing and strategies to mitigate stakeholder burn-out while fostering community ownership, sustained stakeholder engagement and cultural adaptation. These findings offer actionable strategies to policymakers, practitioners and researchers, enabling them to develop resilient, scalable solutions for managing NCDs globally.

## Supplementary material

10.1136/bmjopen-2025-101292 online supplemental file 1

10.1136/bmjopen-2025-101292 online supplemental file 2

10.1136/bmjopen-2025-101292 online supplemental file 3

10.1136/bmjopen-2025-101292 online supplemental file 4

10.1136/bmjopen-2025-101292 online supplemental file 5

## Data Availability

Data are available upon reasonable request.
