# Supplementary material for: Enablers and barriers for scaling up non-communicable disease interventions across diverse global health contexts: a qualitative study using the Consolidated Framework for Implementation Research
Source: BMJ Open. 2025 Dec 10;15(12):e101292. doi: 10.1136/bmjopen-2025-101292 (PMC12699587; doi:10.1136/bmjopen-2025-101292)
Supplement: online supplemental file 1 [file bmjopen-15-12-s001.docx]

***Supplementary file 1: Framework for the document analysis and interviews structured by key factor, CFIR domain and belonging aspects***

| **Key factor** | **CFIR Domain** | **Aspects** | **Findings from the documents** | **Findings in the interview** |
| --- | --- | --- | --- | --- |
| **Intervention** | *Intervention characteristics* | Complexity, compatibility, adaptability, trialability and observability |  |  |
| **Innovation** | *Intervention characteristics* | Relative advantage, evidence strength |  |  |
| **Local context** | *Inner setting* | Examines the organizational context, including culture and resources |  |  |
|  | *Outer setting* | Explores how external factors, such as the socio-political climate and patient needs, impact implementation |  |  |
| **Environment** | *Outer setting* | External factors influencing implementation (political, economic, social factors) |  |  |
| **Local adaptation** | *Intervention characteristics* | Adaptability |  |  |
|  | *Inner setting* | Modifying interventions to fit the specific context in which they are implemented, considering factors such as organizational culture, resources, and individual needs. |  |  |
|  | *Characteristics of individuals* | Knowledge, beliefs, self-efficacy, and individual readiness to adapt the intervention to local settings. |  |  |
| **Stakeholder consultation** | *Process* | Engaging stakeholders throughout the implementation process to facilitate successful adoption and sustainability of the intervention. |  |  |
|  | *Characteristics of individuals* | Communication skills, leadership, and beliefs about the importance of stakeholder involvement in the implementation process. |  |  |
| **Sustainability** | *Intervention characteristics* | Intervention complexity |  |  |
|  | *Inner setting* | Organisational support |  |  |
|  | *Process* | Ongoing stakeholder engagement |  |  |
|  | *Characteristics of individuals* | Persistence, motivation, and long-term commitment to maintaining the intervention. |  |  |
