# Supplementary material for: Enablers and barriers for scaling up non-communicable disease interventions across diverse global health contexts: a qualitative study using the Consolidated Framework for Implementation Research
Source: BMJ Open. 2025 Dec 10;15(12):e101292. doi: 10.1136/bmjopen-2025-101292 (PMC12699587; doi:10.1136/bmjopen-2025-101292)
Supplement: online supplemental file 2 [file bmjopen-15-12-s002.docx]

***Supplementary file 2: Interview guide for follow-up***

**Introduction**

Welcome to the follow-up interview for our study on the enablers and barriers for sustainable scale-up of Non-Communicable Disease (NCD)-related interventions. This interview serves as an essential component of our research, aimed at comprehensively understanding the implementation and scaling-up processes of interventions targeting hypertension and diabetes.

With this interview, we seek to explore the complexities surrounding the long-term sustainability of these interventions. Our overarching goal is to gain insights into how interventions for hypertension and diabetes have been scaled up across diverse contexts. Specifically, we aim to identify the enablers, barriers, and barriers in scaling up these interventions, with a focus on seven key categories: Intervention, Innovation, Local Context, Environment, Local Adaptation, Stakeholder Consultation, and Sustainability.

When we talk about scalability, we refer to the extent to which interventions can be expanded, improved, replicated, or adapted to reach larger populations or additional and/or different settings while maintaining their effectiveness, efficiency, and quality of outcomes. When we talk about sustainability, we refer to the ability of interventions to continue delivering the intended benefits over time, even after initial funding, support, or attention may diminish. In the context of scaling up, sustainability also refers to the ability to maintain the intervention's benefits as it expands to reach larger populations or additional settings.

We have thoroughly collected and analysed data on these key elements related to the project you are or were involved in from various documents. However, this interview presents an opportunity to further clarify, deepen, and complement the information obtained during document analysis.

Please be assured that ethical approval for this study has been obtained, and your consent has been provided for this interview. We prioritise the strict maintenance of data privacy throughout the study, and results will be disseminated through scientific meetings and peer-reviewed journals.

The interview is expected to last approximately 45 minutes (duration depends on how much information is already gathered during the document analysis) and will be recorded for accuracy. Following the interview, the recording will be transcribed for analysis purposes. During this time, we aim to delve into various aspects of the scaling-up process, drawing insights from your experiences and perspectives.

Are there any questions before we start?

1. **Intervention:**

- Can you tell me about the intervention and its implementation? Has the intervention been changed over time? If so, why and what?
- Can you tell me about the project’s scale-up strategy?
- What evidence or rationale supports the effectiveness of the intervention?
- What evidence or rationale supports the scaling up?
- How do you perceive the complexity of the intervention in relation to its implementation and its scaling up?

1. **Innovation:**

- What aspects or approaches of the project do you consider innovative or novel? Why are these aspects or approaches innovative or novel?
- How do you think these innovative elements contributed to the project's goals?
- Which challenges or barriers did you encounter in implementing or scaling-up these innovative aspects?
- How have these innovations impacted the scalability and sustainability of the project?

1. **Local Context:**

- How did the local context influence the implementation and/or scaling up of the intervention?
- What changes in the local context have affected the project's implementation or sustainability?
- Have there been any adaptations made to the project to better fit the local context? If so, what were they, and why were these adaptions made?

1. **Environment:**

- How would you characterise the broader environmental factors affecting the project? (for example, infrastructure, environmental hazards, political and policy)
- Are there political, economic, and/or social factors that influenced the implementation, scalability, and/or sustainability?
- Have there been any changes in the external environment that have impacted the project's implementation or sustainability? If so, what were these changes? And how did this impact the project’s implementation or sustainability?
- How do you anticipate future changes in the environment might affect the intervention/program/project?

1. **Local Adaptation:**

- How has the project been adapted to better fit the needs and preferences of the local stakeholders or community?
- How were factors such as organisational, cultural, and individual needs considered in the project's adaptations?
- Can you provide examples of successful adaptations that have been made during the implementation process?
- Which challenges did you encounter while implementing local adaptations, and how were they addressed?

1. **Stakeholder Consultation:**

- How have stakeholders been involved in the implementation and/or scaling up of the project?
- What mechanisms or strategies have been used to solicit input and feedback from stakeholders?
- What were notable successes in engaging stakeholders throughout the project?
- What were the notable challenges in engaging stakeholders throughout the project?
- How was the scale-up strategy informed by consultation with team members/partners/stakeholders?

1. **Sustainability:**

- How do you envision the sustainability of the project/intervention/program?
- What is needed to ensure the long-term sustainability of the project?
- Have there been any discussions or plans for sustainability built into the project design? If so, what are they? How are stakeholders involved?
- Have any new challenges or unforeseen circumstances emerged during implementation that may impact the project's sustainability?
