# Supplementary material for: Enablers and barriers for scaling up non-communicable disease interventions across diverse global health contexts: a qualitative study using the Consolidated Framework for Implementation Research
Source: BMJ Open. 2025 Dec 10;15(12):e101292. doi: 10.1136/bmjopen-2025-101292 (PMC12699587; doi:10.1136/bmjopen-2025-101292)
Supplement: online supplemental file 3 [file bmjopen-15-12-s003.docx]

**Supplementary file 3: Enablers and barriers with CFIR 2.0 Alignment**

This table presents identified enablers and barriers, their definitions, corresponding CFIR 2.0 constructs and definitions, and overlapping constructs across CFIR domains.

| CFIR Domain | Barrier (B) /Enabler (E) | Definition | CFIR 2.0 Construct & Definition | Overlapping Constructs |
| --- | --- | --- | --- | --- |
| Intervention characteristics | Adaptability (E) | Ability to modify intervention to fit local contexts, infrastructure, and cultural needs | Innovation Adaptability: The innovation can be modified/tailored/refined to fit local context or needs | Individual Characteristics (acceptance, trust) |
|  | Cost-effectiveness (E) | Reducing resource strain through affordable and efficient intervention design | Relative Advantage: Perception that the innovation has more benefit than alternatives | Inner Setting (resource availability) |
|  | Complexity (B) | Resource-intensive interventions requiring extensive training/coordination hinder implementation | Complexity: The perceived difficulty of implementing the innovation | Inner Setting (training, workforce burden) |
|  | Cultural Misalignment (B) | Interventions not aligned with local cultural norms faced resistance | Compatibility: Degree to which the intervention fits with existing values and norms | Outer Setting (cultural context); Individual Characteristics (beliefs) |
| Outer setting | Policy Alignment (E) | Early engagement with policymakers facilitated program integration | External Policy & Incentives: Policies and mandates supporting the innovation | Implementation Process (stakeholder engagement) |
|  | Community-based Organizations (E) | Local organizations embedded interventions into trusted social structures | Cosmopolitanism: The degree to which the organization is networked with others | Inner Setting (partnerships) |
|  | Socio-political Instability (B) | Political instability disrupted timelines and program delivery | External Policy & Incentives: External factors affecting sustainability | Implementation Process (leadership changes) |
| Inner setting | Local Empowerment (E) | Decentralization and autonomy in decision-making enhanced responsiveness | Compatibility: Degree to which innovation fits workflows and values | Individual Characteristics (self-efficacy) |
|  | Capacity Building (E) | Training and skill development improved workforce sustainability | Readiness for Implementation: Access to training and resources | Individual Characteristics (knowledge, self-efficacy) |
|  | Staff Turnover (B) | High turnover disrupted continuity and institutional knowledge | Structural Characteristics: Stability of organizational infrastructure | Implementation Process (sustainability) |
| Individual characteristics | Knowledge Gaps (B) | Inadequate training limited capacity to manage NCDs | Knowledge & Beliefs about the Intervention | Inner Setting (capacity building) |
|  | Cultural Resistance (B) | Skepticism about new health programs hindered uptake | Knowledge & Beliefs about the Innovation | Intervention Characteristics (cultural misalignment) |
| Implementation process | Stakeholder Engagement (E) | Early and continuous involvement of stakeholders ensured buy-in | Engaging: Involving appropriate stakeholders in the process | Outer Setting (policy alignment); Inner Setting (partnerships) |
|  | Stakeholder Burnout (B) | Overwhelming demands reduced engagement over time | Engaging: Maintaining stakeholder involvement | Inner Setting (workforce burden) |
|  | Short Project Timeframes (B) | Limited ability to evaluate long-term impacts | Planning: Developing strategies to execute implementation | Outer Setting (funding constraints) |
