# Supplementary material for: Enablers and barriers for scaling up non-communicable disease interventions across diverse global health contexts: a qualitative study using the Consolidated Framework for Implementation Research
Source: BMJ Open. 2025 Dec 10;15(12):e101292. doi: 10.1136/bmjopen-2025-101292 (PMC12699587; doi:10.1136/bmjopen-2025-101292)
Supplement: online supplemental file 4 [file bmjopen-15-12-s004.docx]

**Supplementary file 4: Mapping of enablers and barriers to WHO health system building blocks**

This table demonstrates how identified CFIR enablers and barriers align with WHO’s six health system building blocks, with representative quotes/examples, and overlapping constructs.

| **WHO Building Block** | **CFIR Domain** | **Barrier (B) /Enabler (E)** | **Key Quote/Example** | **Overlapping Constructs** |
| --- | --- | --- | --- | --- |
| **Service Delivery** | Intervention Characteristics | Adaptability (E) | Tailoring interventions to local service structures improved integration and sustainability. | Inner Setting (local adaptation) |
|  | Implementation Process | Stakeholder Engagement (E) | Collaboration with community leaders improved uptake of dietary interventions. | Outer Setting (policy alignment) |
|  | Intervention Characteristics | Complexity (B) | Resource-intensive designs strained delivery capacity. | Inner Setting (resource limitations) |
| **Health Workforce** | Inner Setting | Capacity Building (E) | Training improved confidence of health workers to manage NCDs. | Individual Characteristics (knowledge, self-efficacy) |
|  | Individual Characteristics | Knowledge Gaps (B) | Healthcare workers lacked training for complex NCD cases. | Inner Setting (operational issues) |
|  | Inner Setting | Staff Turnover (B) | Frequent turnover disrupted continuity of care. | Implementation Process (sustainability) |
| **Health Information Systems** | Inner Setting | Integration of Digital Tools (E) | Digital platforms maintained healthcare delivery during COVID-19 lockdowns. | Outer Setting (digital adoption) |
|  | Individual Characteristics | Low Digital Literacy (B) | Low digital literacy hindered use of mobile health apps. | Outer Setting (infrastructure barriers) |
| **Medicines & Technologies** | Outer Setting | Resource Limitations (B) | COVID-19 redirected resources away from NCD programs. | Intervention Characteristics (cost-effectiveness) |
|  | Outer Setting | Regulatory Barriers (B) | Weak regulations and bureaucratic delays slowed scale-up. | Implementation Process (planning) |
| **Financing** | Intervention Characteristics | Cost-effectiveness (E) | Innovative integration reduced costs, enabling sustainability. | Inner Setting (resources) |
|  | Outer Setting | Resource Limitations (B) | Funding constraints led to halted or delayed activities. | Inner Setting (funding constraints) |
| **Leadership/Governance** | Outer Setting | Policy Alignment (E) | Early policymaker involvement ensured integration into national guidelines. | Implementation Process (stakeholder engagement) |
|  | Outer Setting | Socio-political Instability (B) | Civil unrest and coups disrupted timelines and commitments. | Implementation Process (leadership changes) |
|  | Implementation Process | Stakeholder Burnout (B) | Fatigue from multiple programs reduced engagement. | Inner Setting (workforce burden) |
