# Supplementary material for: Enablers and barriers for scaling up non-communicable disease interventions across diverse global health contexts: a qualitative study using the Consolidated Framework for Implementation Research
Source: BMJ Open. 2025 Dec 10;15(12):e101292. doi: 10.1136/bmjopen-2025-101292 (PMC12699587; doi:10.1136/bmjopen-2025-101292)
Supplement: online supplemental file 5 [file bmjopen-15-12-s005.docx]

***Supplementary file 5: Overview of number of enablers and barriers per CFIR domain spread over projects and regions***

|  | **Enablers** | **Projects** | AFR | AMR | SEAR | EUR | WPR | **Barriers** | **Projects** | AFR | AMR | SEAR | EUR | WPR |
| --- | --- | --- | --- | --- | --- | --- | --- | --- | --- | --- | --- | --- | --- | --- |
| Intervention characteristics | Adaptability | 4 | ✓ | ✓ | ✓ | ✓ |  | Cultural misalignment | 2 | ✓ | ✓ |  |  |  |
|  | Cost-effectiveness and streamlined processes | 3 | ✓ | ✓ |  | ✓ |  | Complexity of interventions | 2 | ✓ |  |  | ✓ |  |
|  | Innovative approaches | 3 | ✓ | ✓ |  | ✓ |  | Systematic barriers | 2 | ✓ |  | ✓ |  |  |
|  |  |  |  |  |  |  |  | Limited flexibility | 2 |  | ✓ | ✓ |  |  |
|  |  |  |  |  |  |  |  | Misalignment community initiatives and primary healthcare services | 2 | ✓ | ✓ |  |  |  |
| Inner setting | Effective decentralization | 5 |  |  | ✓ |  |  | Resource limitations and funding constraints | 7 | ✓ | ✓ |  |  |  |
|  | Local empowerment | 5 |  |  | ✓ |  |  | Operational issues | 5 |  |  | ✓ |  |  |
|  | Strong partnerships with local stakeholders | 4 | ✓ | ✓ |  |  |  | Cultural resistance | 4 | ✓ | ✓ |  |  |  |
|  | Integration of digital tools | 4 | ✓ |  |  |  |  | Technological constraints | 4 | ✓ |  | ✓ |  |  |
|  | Local adaptation | 6 |  |  | ✓ |  |  |  |  |  |  |  |  |  |
|  | Capacity building | 4 | ✓ |  |  |  |  |  |  |  |  |  |  |  |
| Outer setting | Role of community-based organizations | 4 | ✓ | ✓ |  |  |  | Regulatory barriers and bureaucratic hurdles | 3 | ✓ |  | ✓ | ✓ |  |
|  | Alignment with national policies | 4 | ✓ | ✓ |  | ✓ |  | Resource limitations | 5 | ✓ |  | ✓ |  |  |
|  | Supportive local governance | 4 | ✓ | ✓ | ✓ |  |  | Socio-political instability | 3 | ✓ | ✓ | ✓ |  |  |
|  | Digital health adoption | 4 | ✓ | ✓ | ✓ | ✓ |  | Infrastructural deficiencies | 2 |  |  | ✓ |  |  |
|  |  |  |  |  |  |  |  | Cultural resistance | 4 | ✓ |  | ✓ |  |  |
|  |  |  |  |  |  |  |  | Fragmented health systems | 3 | ✓ | ✓ |  |  |  |
| Individual characteristics | Targeted training and education | 4 | ✓ |  | ✓ |  |  | Knowledge gaps among health workers | 5 | ✓ | ✓ |  |  |  |
|  | Cultural adaptation for acceptance of interventions | 4 | ✓ | ✓ | ✓ |  |  | Resistance to entrenched cultural beliefs | 3 | ✓ | ✓ | ✓ |  |  |
|  | Use of technology | 3 | ✓ | ✓ |  | ✓ |  | Skepticism about new health programs | 3 | ✓ | ✓ | ✓ |  |  |
|  |  |  |  |  |  |  |  | Socioeconomic disparities | 5 | ✓ | ✓ | ✓ |  | ✓ |
|  |  |  |  |  |  |  |  | Low health digital literacy | 5 | ✓ |  | ✓ |  |  |
| Implementation process | Stakeholder engagement | 5 | ✓ | ✓ | ✓ |  |  | Changes in leadership | 4 | ✓ | ✓ | ✓ |  |  |
|  | Continuous incorporation of stakeholder feedback | 2 | ✓ | ✓ |  |  | ✓ | Short project timeframes | 3 | ✓ | ✓ |  |  |  |
|  | Creating co-ownership | 3 | ✓ | ✓ |  |  |  | Systematic barriers | 4 |  |  | ✓ | ✓ |  |
|  | Ongoing monitoring and evaluation | 4 | ✓ |  |  | ✓ | ✓ | Complexity of coordinating multi-sectoral involvement | 4 | ✓ |  | ✓ |  |  |
|  |  |  |  |  |  |  |  | Stakeholder burnout | 3 | ✓ | ✓ |  |  |  |
