# Supplementary material for: Enablers and barriers for scaling up non-communicable disease interventions across diverse global health contexts: a qualitative study using the Consolidated Framework for Implementation Research
Source: BMJ Open. 2025 Dec 10;15(12):e101292. doi: 10.1136/bmjopen-2025-101292 (PMC12699587; doi:10.1136/bmjopen-2025-101292)
Supplement: Supplementary file 6 [file bmjopen-2025-101292.reviewer_comments.pdf]

PEER REVIEW HISTORY

BMJ Open publishes all reviews undertaken for accepted manuscripts. Reviewers are asked to complete a checklist review form and are provided with free text boxes to elaborate on their assessment. These free text comments are reproduced below.

ARTICLE DETAILS

Title (Provisional)

Enablers and barriers for scaling up non-communicable disease interventions across diverse global health contexts: a qualitative study using the Consolidated Framework for Implementation Research

Authors

Pardoel, Zinzi; Folkertsma, I.; Ramani-Chander, Anusha; Thrift, Amanda G; Joshi, Rohina; Bandurek, I.; van Olmen, Josefien; Shrestha, Abha; Delobelle, Peter; Webster, Jacqui; Stehr, L.; Hirschhorn, Lisa R; Koot, Jaap; Alma, M.A.; Wouters, Edwin; Maharani, Asri; sujarwoto, sujarwoto; Siddiqi, Kamran; Widyaningsih, Vitri; Miranda, J. Jaime; Zhang, Puhong; Liu, Hueiming; Theilmann, Michaela; Probandari, Ari; Rawal, Lal B.

VERSION 1 - REVIEW

**Reviewer**	**1**
**Name**	**Laur, Celia**
**Affiliation**	**Women's College Hospital**
**Date**	**28-Jun-2025**
**COI**	**None**

Enablers and barriers for scaling up non-communicable disease interventions across diverse global health contexts: insights from using the Consolidated Framework for Implementation Research

This multi-method study aims to identify key barriers and enablers to the sustainability and scalability of non-communicable disease interventions across diverse global health contexts. Fifteen projects, aimed at scaling across 18 countries, were studied through document analysis and stakeholder interviews. This is a comprehensive study that applies a well-known implementation framework (CFIR 2.0) to guide and structure the results. Although there are many changes listed here, many are more for clarification and to strengthen your results.

Major Changes

A table with your named barriers and enablers, your definition for each, their alignment with the relevant CFIR 2.0 construct name(s) (since you are not using the direct CFIR 2.0 construct names and definitions), and a key quote/example would strengthen your results. If possible, including a

column of when the same theme connects with more than one construct would be useful to highlight the overlap you emphasize. In line with reporting guidelines, further quotes or examples to go with each barrier/enabler, not just each overarching domain, would strengthen the results. As the supporting information may be from an interview or document analysis, it might be an example presented from one of the documents, not just interview quotes.

The Table could look something like this:

**CFIR Domain**	**Identified Barrier (B) /Enabler (E)**	**Definition**	**Key quote/ example**	**Corresponding CFIR 2.0 Construct(s) & Definition(s)**	**Overlapping Constructs**
Intervention Characteristics	Adaptability (E)	*How well interventions can be modified to fit local contexts, healthcare infrastructure, and cultural needs.*		*Innovation Adaptability:* The innovation can be modified, tailored, or refined to fit local context or needs	Individual Characteristics
	Cost-Effectiveness (E)				

Your second objective does not seem to be directly answered by this paper. Although the discussion includes the figure and mentions mapping, there are no methods for how this mapping was completed, nor do there appear to be results of this mapping. Is the Figure in the discussion the original WHO building blocks, or mapped? It's hard to tell. I understand the benefit to include this comparison, however if it remains as an objective, further detailed methods and results are needed about this mapping process. If it's removed as an objective, it could be reworded in the discussion a more of a reflection regarding how this work compares with the building blocks. Further clarity is needed in the Figure as the terms used don't directly match your results.

Minor Changes

- Please confirm if you followed the CFIR 2.0 from the original Damschroder et al. 2022, or the image in Figure 1. These are not the same as The Centre for Implementation (TCI) has changed some of the constructs. If you followed Damschroder 2022, there is now a visual that accompanies the update and more directly aligns with the paper than the TCI version.

- Throughout the results, be sure to emphasize how the results relate to scalability. Although there is a lot of overlap, the unique aspect of this work is to focus on the barriers and enablers to scalability, however some results still seem to focus on implementation, which could be interpreted as initial implementation, compared to implementation in new settings as part of the scaling process. Obviously there is a lot of overlap and nuance here but emphasizing your focus on scalability and sustainability through the results will make this more clear and unique.

- Near line 145, you mention the projects were "nearing or completing scaling-up". Can you please provide more detail on how this was determined and the level of the scaling that had occurred?
  Does this mean they reached a pre-specified target? If so, what was the target? Was it the same across sites? With the lack of standardization about what is meant by "scale", this detail would provide valuable information to help contextualize your results.

- For Table 1, are WHO regions the original region, or the regions covered at the time of the data collection? Did programs scale start in one and scale to multiple countries?

- Please state if the interviewers had a prior relationship with any of the stakeholders/interviewees.

- Line 192 - It is unclear why there is a "(1794)" after the word Results

- Please include rationale for not using CFIR 2.0 construct names directly. Is this to make it more applicable, understandable, and targeted to your context? You don't need to use the direct CFIR terms (especially if you develop the Table recommended above), but having this justification for using different terminology in your methods is recommended.

- Near line 204, how was cost effectiveness presented in other studies? It currently read as though this was only found in one study which I assume is not the case.

- Near line 206, further description of the "innovation" enabler is needed as the current description is unclear.

- Near line 210, you mention complexity of the intervention as a barrier to implementation, however as you are looking at scalability, is this a barrier to initial implementation, or to continued implementation in multiple settings, or sustainability?

- Near line 218-219 it mentions the disconnect in the intervention design and the communities lived experience. Was this also the case with initial implementation, or was this a new challenge when trying to scale the intervention? The statement regarding the need for cultural sensitivity could be strengthened by acknowledging if this is an ongoing need, not just at the beginning of implementation, but needs to be considered throughout implementation, and while sustaining and scaling.

- Line 248 - It is unclear where Quote 3 aligns? The role of community health workers seems important, but not sure which barrier/enabler this quote supports?
- Line 314 – you mention economic factors limiting access to healthy food etc.. Is this specifically income etc., for an individual/family? On first reading I interpreted economic factors as more a system-level concern, so further explanation of this section would be beneficial.

- Line 324 – as you're focused on stainability and scale, was the stakeholder engagement needed at later stages as well, as it currently only mentions planning and execution.

- Line 380-381 – The statement about how simplifying an intervention may inadvertently dilute its core. Did you find that in your studies? This is an interesting point and tying it more directly to your results will deepen this reflection.

- Is any more quantitative data available (from other publications) that can be referenced to help contextualize some of the effectiveness of the interventions studied? Do you know if you are exploring cases that have had the intended benefit? Are you only looking at "successful" cases that had an impact, or it is learnings from any case which may or may not kept the intended benefits as it scaled? If you don't know if the interventions were having the intended benefit, this needs to be acknowledged in the limitations.

- Line 391 – In the previous paragraph you mention that Greenhalgh highlights stakeholder

burnout as a critical barrier, yet in Line 391 you mention this as a “novel” finding. I’m also not sure that the interplay of financial and system components is novel either as I believe that has been shown many times before? I suggest rewording this section.

- Line 395 – Can you please explain further how the systems thinking approach can be applied and how that relates to your work?

- Line 454 – I think I understand what you mean, but I think this section needs to be rephrased, as even when an intervention is designed to align with health system priorities, it can still encounter barriers, particularly from those that are outside the control of those running the intervention. For example, just because the intervention aligns with health system priorities, does not mean political instability wont still be a barrier.

- Line 489 – This last line is the first mention of “co-design”. Maybe worth rewording to more directly align with the rest of the paper rather than introducing a new concept.

**Reviewer**	**2**
**Name**	**Hanekom, Susan**
**Affiliation**	**Stellenbosch University, Department of Physiotherapy**
**Date**	**31-Jul-2025**
**COI**	**None**

Thank you for the opportunity to review this interesting paper: "Enablers and barriers for scaling up non-communicable disease interventions across diverse global health contexts: insights from using the Consolidated Framework for Implementation Research"

Mayor concern:

A danger of working with such a large database is that information becomes redundant without adding new insights. The authors appear to be are aware of the danger and highlight which new insights the paper is adding. e.g. Building on these insights and gaps identified in the preliminary research,18,21 this follow-up study

explores opportunities to advance previous findings. Will be beneficial for reader if the authors could contextualise the study. How are the three papers linked? That will the provide an opportunity to clearly define tools that were developed to extract and analyse data.

It is not clear why you used the CFIR framework. Please motivate - did you find the WHO building block structure more beneficial? Please explain

The limitation identified by authors regarding stakeholder identification is VERY important. Does not allow a true evaluation of the individual factors in CFIR. Supports question of which new insights are offered

Minor concerns:

Some areas of the methodology also include results for example the documents submitted for analysis. Table 1: results

It is not clear if interviews were linked to document review of the specific project?E.g "The interviews focused on underreported or unclear information from documentation related..."

Authors refer to different systems involved in sustainability but then only refer to "health care systems". Which other systems did authors identify?

Not sure on which data the following strong statement is based: "Scaling-up initiatives must embed a systems-thinking approach into their design"

Please differentiate between "co-design" of intervention and "contextualize" intervention. Which concept emerged from your data?

I was unable to locate the paper: Agyepong IA, Adjei S. Addressing the NCD burden in LMICs: The role of the Global Alliance for Chronic Diseases. Health Policy Plan. 2022;37(2):160–7. Please check also all references

---

VERSION 1 - AUTHOR RESPONSE

Reviewer 1

We thank the first reviewer for the comprehensive review and constructive feedback, which improved the manuscript.

Major comments:
Comment 1. Table with barriers/enablers, definitions, CFIR 2.0 constructs, quotes, overlaps.
[Response] Based on the reviewer's comment and suggested table, we have developed Supplementary file 3, which presents each barrier/enabler, our definition, the aligned CFIR 2.0 construct(s), and overlapping constructs. We did not include quotes in this table, as these are already integrated in the Results to illustrate each barrier/enabler. Adding them again would have caused redundancy and reduced readability. The supplementary file strengthens the results by improving transparency and alignment with CFIR terminology. In the methods section we have added the following text in lines 232-233: "*To enhance transparency and alignment with the original framework, supplementary file 3 provides the corresponding CFIR 2.0 construct names and definitions for each barrier and enabler.*"
In addition, following the reviewer's suggestion, we also reviewed whether some of the interview quotes could be replaced with quotes from the document analysis. We replaced quote 4, where a document quote was clearly more suitable. For the other quotes, we did not identify document excerpts that were more appropriate than the existing interview quotes.

Comment 2. Mapping to WHO building blocks unclear.
[Response] Thank you for this suggestion. We now added a new methods subsection on mapping in lines 233-240: "*To address our secondary objective, we also systematically mapped the identified enablers and barriers from CFIR domains to the World Health Organization's (WHO)*

six health system building blocks: service delivery, health workforce, health information systems, access to essential medicines and technologies, financing, and leadership/governance. This deductive mapping approach was conducted in Excel, whereby each CFIR-coded barrier or enabler was assigned to one or more relevant WHO building blocks. Where enablers or barriers aligned with more than one building block, they were noted as overlapping to highlight cross-cutting relevance (Supplementary file 4)."

Moreover, we have added a results subsection on alignment in lines 424-444:

Mapping to WHO health system building blocks

The mapping exercise demonstrated a strong alignment between CFIR-identified barriers/enablers and the WHO building blocks (Figure 3; Supplementary file 4).

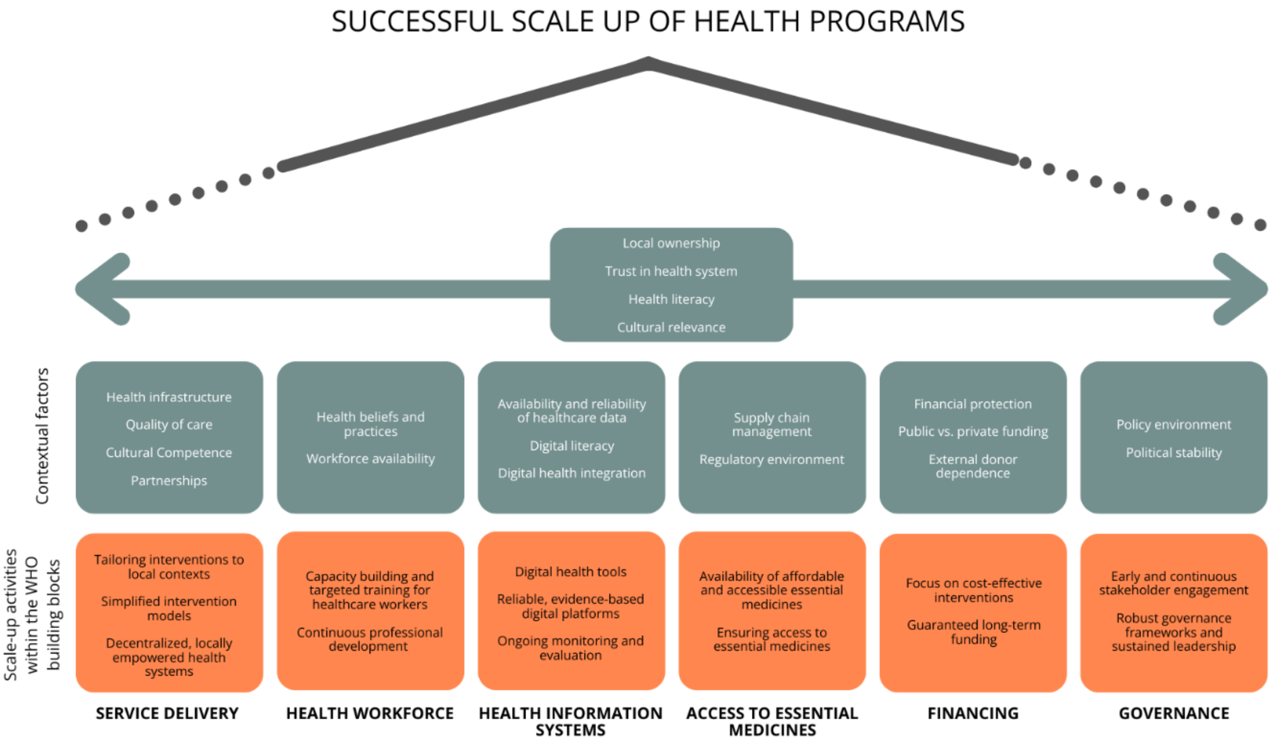

Figure 3 Key enablers for scaling up sustainable health programs: alignment with the WHO's six building blocks of health systems[12]

Service delivery was most influenced by factors such as adaptability and stakeholder engagement, each of which facilitated integration of interventions into existing services, while greater complexity of interventions diminished the capacity for their delivery. Health workforce mapping highlighted capacity building and targeted training as critical for sustainability, contrasted by knowledge gaps and staff turnover, which undermined continuity of care. Moreover, health information systems benefitted from integration of digital tools that supported care during the COVID-19 pandemic, but faced barriers including low digital literacy and infrastructural challenges. Further, medicines and technologies were linked to resource limitations and regulatory barriers, especially in contexts where weak systems delayed scale-up or diverted supplies. Financing overlapped with intervention characteristics, where cost-effectiveness supported sustainability, but funding constraints and short project timeframes limited scalability. Lastly, leadership and governance aligned strongly with policy alignment and supportive local governance, which facilitated long-term embedding of programs. However, socio-political instability and stakeholder burnout reflected fragility in sustaining momentum. Several cross-

cutting factors, particularly adaptability, stakeholder engagement, and resource limitations, spanned multiple building blocks, underlining their systemic influence on scale-up efforts."

Further we have revised the following text in the discussion, lines 464-470: *Our findings not only highlight barriers and enablers across CFIR domains but also demonstrate how these factors align with the WHO's six building blocks of health systems. As illustrated in Figure 3 and detailed in supplementary file 4, scalability was influenced by enablers such as adaptability, stakeholder engagement, and cost-effectiveness, while barriers such as complexity, resource limitations, and socio-political instability spanned multiple system components. This mapping provides an integrated perspective that connects implementation science with global health system frameworks, strengthening the applicability of our results for policy and practice.*
Lastly, we have clarified the figure 3 caption, and added Supplementary file 4 with a table clarifying the full mapping matrix. This ensures clarity on how the mapping was conducted and interpreted.

Minor comments:

Comment 3. Please confirm if you followed the CFIR 2.0 from the original Damschroder et al. 2022, or the image in Figure 1. These are not the same as The Centre for Implementation (TCI) has changed some of the constructs. If you followed Damschroder 2022, there is now a visual that accompanies the update and more directly aligns with the paper than the TCI version.
[Response] Thank you for this important clarification. We confirm that we followed the CFIR 2.0 framework as published by Damschroder et al. (2022). To ensure alignment with the peer-reviewed update, we have replaced Figure 1 with the original CFIR 2.0 visual published by Damschroder et al. (2022). We also revised the methods (lines 144–147) to clarify:
"To ensure alignment with the peer-reviewed update, we used the original CFIR 2.0 visual published by Damschroder et al. (2022) (Figure 1), which illustrates the five domains and corresponding constructs. This framework provided a structured lens for identifying and analyzing barriers and enablers of scalability across projects."

Comment 4. Throughout the results, be sure to emphasize how the results relate to scalability. Although there is a lot of overlap, the unique aspect of this work is to focus on the barriers and enablers to scalability, however some results still seem to focus on implementation, which could be interpreted as initial implementation, compared to implementation in new settings as part of the scaling process. Obviously, there is a lot of overlap and nuance here but emphasizing your focus on scalability and sustainability through the results will make this clearer and more unique.
[Response]We revised the Results throughout to explicitly emphasize scalability and sustainability. For example, lines 258-260 are revised to: "Innovation, i.e., introducing new methods in the context of the project for managing NCDs or integrating services, was another enabler *of scalability, as novel approaches supported adaptation across contexts and long-term embedding within health systems."* Similar adjustments were made throughout the results, for example in the sections on complexity (lines 271-273) and cultural misalignment (lines 279–283), as copied below.

Lines 271-273: "In contrast, the **complexity of interventions** was a significant barrier *to scalability and sustainability, whereby resource-intensive interventions requiring extensive coordination or training created challenges for expansion into multiple settings and for maintaining fidelity over time."*

Lines 282-288: *"Disconnects between intervention design and communities' lived experiences emerged as barriers not only during initial implementation but also as projects expanded into new settings. Cultural sensitivity is an ongoing need, essential from the outset and throughout*

sustainability to ensure acceptance and engagement. Stakeholders emphasized the need for culturally sensitive interventions to ensure acceptance, sustainability, and scalability. While aligning with cultural contexts is vital, they noted that achieving broader health goals may sometimes necessitate gradual adjustments to local practices, reinforced through community engagement and ongoing collaboration to sustain scale-up."

In addition, we clarified in the introduction how we conceptualize scale-up, to make our framing explicit. In line with earlier scale-up research, we describe scale-up in terms of expansion, coverage, and institutionalization. We acknowledge that expansion may overlap with implementation activities, but we highlight that scale-up extends beyond initial delivery to embed interventions more broadly and sustainably within health systems. We have added the following text in lines 122-126: "*In line with earlier scale-up research [24], we conceptualize scale-up in terms of expansion, coverage, and institutionalization. We recognize that expansion may overlap with implementation activities, but highlight that scale-up extends beyond initial delivery to embed interventions more broadly and sustainably within health systems."*

Comment 5. Near line 145, you mention the projects were "nearing or completing scaling-up". Can you please provide more detail on how this was determined and the level of the scaling that had occurred? Does this mean they reached a pre-specified target? If so, what was the target? Was it the same across sites? With the lack of standardization about what is meant by "scale", this detail would provide valuable information to help contextualize your results.
[Response] Thank you for this important point. We have clarified how we determined that projects were "nearing or completing scale-up." In this study, we included only projects that were considered to be 'nearing or completing scale-up'. Of the original 27 GACD-funded implementation research projects, 6 were excluded because they had not yet reached this stage. Projects were considered to be 'nearing or completing scale-up' if they had reached key expansion milestones beyond pilot implementation, such as moving from single-site pilots to multiple districts/provinces, formal adoption within national or regional health systems, or integration into policy frameworks. We note that no uniform pre-specified target for scale-up existed across projects; instead, milestones varied depending on context, intervention design, and health system priorities. This clarification has been added in the Methods (lines 161–169): "*In this study, we included only projects that were considered to be 'nearing or completing scale-up'. Of the original 27 GACD-funded implementation research projects, 6 were excluded because they had not yet reached this stage. Projects were considered to be 'nearing or completing scale-up' if they had reached key expansion milestones beyond initial pilot implementation. These milestones included extending interventions to multiple districts or provinces, formal adoption within regional or national health systems, or integration into national policy frameworks. Because of contextual differences, no single pre-specified target for scale-up was applied across projects; rather, the definition of scale-up was based on progress toward embedding interventions beyond pilot phases within health systems."*

Comment 6. For Table 1, are WHO regions the original region, or the regions covered at the time of the data collection? Did programs scale start in one and scale to multiple countries?
[Response] Thank you for this helpful request for clarification. The WHO regions presented in Table 1 reflect the geographic coverage of the interventions at the time of data collection, which in some cases extended beyond the original pilot region. Several projects began in one country and later expanded to additional sites or countries, while others remained within the original WHO region. We have added text under Table 1 to make this explicit. Added text under Table 1, from line 191: "*WHO regions presented in this table reflect the geographic coverage of the interventions at the time of data collection. In some cases, projects expanded from their original site into multiple countries or regions, while others remained within the initial WHO region where they were piloted.*"

Comment 7. Please state if the interviewers had a prior relationship with any of the stakeholders/interviewees.

[Response] Thank you for this important clarification request. We confirm that the interviewers did not have any prior relationship with the stakeholders or interviewees before the study commenced. We have added this detail to the Methods section under Sample and data collection, line 216: "*who had no prior relationships with any of the interviewed stakeholders before the study,"*

Comment 8. Line 192 - It is unclear why there is a "(1794)" after the word Results
[Response] Thank you for noticing this. The "(1794)" was a typographical error and has been removed from the manuscript.

Comment 9. Please include rationale for not using CFIR 2.0 construct names directly. Is this to make it more applicable, understandable, and targeted to your context? You don't need to use the direct CFIR terms (especially if you develop the Table recommended above), but having this justification for using different terminology in your methods is recommended.
[Response] Thank you for this suggestion. We agree that it is important to explain our choice of terminology. We intentionally used plain-language barrier and enabler labels instead of the original CFIR construct names to make our findings more accessible, applicable, and understandable for a broad global health audience, including practitioners and policymakers who may not be familiar with CFIR terminology. To ensure transparency, we developed Supplementary file 3, where each barrier/enabler is explicitly aligned with the corresponding CFIR 2.0 construct(s). This approach balances accessibility with methodological rigor. We have added the following clarification to the methods, lines 230-233: "*For reporting purposes, we applied plain-language labels to the identified barriers and enablers to ensure accessibility for global health practitioners and policymakers less familiar with CFIR terminology. To maintain transparency and alignment with the original framework, Supplementary file 3 provides the corresponding CFIR 2.0 construct names and definitions for each barrier and enabler.*"

Comment 10. Near line 204, how was cost effectiveness presented in other studies? It currently read as though this was only found in one study which I assume is not the case.
[Response] Thank you for this observation. We clarified that cost-effectiveness was reported across multiple projects in line 257-259: "Moreover, **cost-effectiveness** facilitated scalability by reducing resource strain, *as seen in multiple projects. For example, in the region of the Americas (AMR), the integration of an innovative program reduced costs and complexity, making it feasible for long-term sustained scale-up."*

Comment 11. Near line 206, further description of the "innovation" enabler is needed as the current description is unclear.
[Response] Thank you for pointing this out. We expanded the description of the innovation enabler and provided examples to make it clearer and more concrete in lines 261-266: "**Innovation**, *i.e., the introduction of new approaches in the projects for managing NCDs or integrating services, was another enabler of scalability, as novel methods supported adaptation across contexts and long-term embedding within health systems. Examples included the use of digital health tools to extend care delivery, community-based delivery models to increase reach, and cross-sectoral collaborations to integrate NCD care into existing health and social services. Such innovations enhanced adaptability and facilitated scalability across diverse contexts."*

Comment 12. Near line 210, you mention complexity of the intervention as a barrier to implementation, however as you are looking at scalability, is this a barrier to initial implementation, or to continued implementation in multiple settings, or sustainability?
[Response] In line with our revisions responding to Comment 4, we updated the Results to emphasize that complexity was primarily a barrier to scalability and sustainability across multiple settings, rather than only to initial implementation. We have copied our changes below for convenience:
Lines 271-273: "In contrast, the **complexity of interventions** was a significant barrier *to scalability and sustainability, whereby resource-intensive interventions requiring extensive coordination or*

training created challenges for expansion into multiple settings and for maintaining fidelity over time."

Comment 13. Near line 218-219 it mentions the disconnect in the intervention design and the communities lived experience. Was this also the case with initial implementation, or was this a new challenge when trying to scale the intervention? The statement regarding the need for cultural sensitivity could be strengthened by acknowledging if this is an ongoing need, not just at the beginning of implementation, but needs to be considered throughout implementation, and while sustaining and scaling
[Response]Thank you for this thoughtful suggestion. We have added text to clarify that cultural misalignment posed challenges both during initial implementation and more prominently during scalability, and that cultural sensitivity is an ongoing requirement throughout scale-up and sustainability, in lines 282-285: *Disconnects between intervention design and communities' lived experiences emerged as barriers not only during initial implementation but also as projects expanded into new settings. Cultural sensitivity is an ongoing need, essential from the outset and throughout sustainability to ensure acceptance and engagement."*

Comment 14. Line 248 - It is unclear where Quote 3 aligns? The role of community health workers seems important, but not sure which barrier/enabler this quote supports?
[Response] Thank you for this observation. We revised the Results to make clear that Quote 3 aligns with the enabler 'role of community-based organisations' under the Outer setting domain. Specifically, it illustrates how community health workers, often engaged through community-based organisations, were empowered to participate actively and given a voice, thereby strengthening ownership and contributing to scalability. We have now added the following text (new text in *italics*) in lines 301-307 (we have also copied below the preceding two sentences, to provide context to this added text): "Within the domain of the *Outer setting*, **enablers** included the crucial **role of community-based organizations**. These organizations mobilized hard-to-reach populations and integrated interventions into local social structures. For instance, a community-based organization in the South-East Asian Region (SEAR) was essential in connecting older populations with health programs, ensuring that interventions were embedded in social systems that people trust and engage with regularly. *Quote 3 further illustrates this enabler, highlighting how community health workers, when given a voice and space to participate actively, strengthened ownership and made interventions work effectively."*

Comment 15. Line 314 – you mention economic factors limiting access to healthy food etc.. Is this specifically income etc., for an individual/family? On first reading I interpreted economic factors as more a system-level concern, so further explanation of this section would be beneficial.
[Response] Thank you for this observation. We have now clarified that the economic factors described here primarily referred to household- and individual-level financial constraints (e.g., limited income restricting access to healthy food and medicines), while also noting the broader system-level implications for scalability. We added the following text in lines 388-391: "*particularly household- and individual-level financial constraints such as limited income, restricted access to healthy foods and medicines, complicating the promotion of healthier lifestyles. These constraints not only affected individual participation but also posed broader challenges to the scalability and sustainability of interventions in resource-limited settings.*"

Comment 16. Line 324 – as you're focused on stainability and scale, was the stakeholder engagement needed at later stages as well, as it currently only mentions planning and execution.
[Response] We have now clarified that stakeholder engagement was critical not only in planning and execution but also in the later stages of scale-up and sustainability, ensuring continued ownership, adaptation, and alignment with evolving system needs. We revised the text in lines 399-402: "Related to this, we found continuous incorporation of stakeholder feedback *throughout the interventions' planning, execution and* later stages of scale-up, *was an enabler*

ensuring long-term sustainability and adaptability by allowing interventions to be adapted in real time to meet local needs and barriers."

Comment 17. Line 380-381 – The statement about how simplifying an intervention may inadvertently dilute its core. Did you find that in your studies? This is an interesting point and tying it more directly to your results will deepen this reflection.
[Response] Thank you for raising this point. We did not find direct evidence in our dataset that stakeholders experienced the dilution of core intervention components when simplifying for scalability. However, we agree that this tension is well recognized in the literature and highly relevant to the interpretation of our findings on intervention complexity. To clarify, we have revised the discussion to make explicit that this was not reported in our study but reflects broader evidence from implementation science. We also highlighted models of cultural adaptation that can help address this challenge by safeguarding core elements while allowing flexibility for scale-up. We revised this in the lines 502-509: "Intervention complexity was identified as a barrier, particularly in LMICs, where decentralized, locally empowered systems are essential for community-responsive care. This finding aligns with Peters et al., 2023,[29] and Gilson et al., 2014,[30] who stressed the importance of local flexibility in overcoming systemic barriers. *While the participants in this study did not report this explicitly, the literature highlights an inherent tension: simplifying an intervention to enhance scalability may inadvertently dilute its evidence-based core. Models of cultural adaptation, such as Bernal and Sáez-Santiago's ecological validity framework (2006),[31] provide a way to safeguard core components while addressing local sociocultural dynamics*."

Comment 18. Is any more quantitative data available (from other publications) that can be referenced to help contextualize some of the effectiveness of the interventions studied? Do you know if you are exploring cases that have had the intended benefit? Are you only looking at "successful" cases that had an impact, or it is learnings from any case which may or may not kept the intended benefits as it scaled? If you don't know if the interventions were having the intended benefit, this needs to be acknowledged in the limitations.
[Response] Our study was designed as a qualitative analysis of scale-up processes, and not all projects had published effectiveness data at the time of analysis. We therefore focused on identifying barriers and enablers to scalability, regardless of whether interventions had demonstrated the intended quantitative benefits. We acknowledge as a limitation that we did not assess effectiveness directly, and that some cases may not have retained their intended benefits during scale-up. We have now added the following text in lines 633-637 to include this limitation: "*Our study was designed as a qualitative exploration of scale-up processes rather than an evaluation of intervention outcomes. While some included projects have quantitative effectiveness data reported elsewhere[33-39], we did not systematically assess effectiveness, and it remains unclear whether all projects maintained their intended benefits during scale-up. Therefore, our findings should be interpreted as insights into scale-up processes rather than assessments of intervention impact.*"

Comment 19. Line 391 – In the previous paragraph you mention that Greenhalgh highlights stakeholder burnout as a critical barrier, yet in Line 391 you mention this as a "novel" finding. I'm also not sure that the interplay of financial and system components is novel either as I believe that has been shown many times before? I suggest rewording this section.
[Response] We agree that both stakeholder burnout and the interplay between financing and system-level challenges have been described in prior literature and therefore should not be presented as novel findings. We have reworded this section to clarify that our contribution lies in reinforcing and contextualizing these well-documented barriers within the specific setting of LMIC scale-up efforts. The revised text in lines 520-529 are copied below: "*Stakeholder burnout and the interplay between financing and system components, while recognized in prior research, were strongly reinforced by*

our findings, particularly in LMIC contexts where these challenges were compounded by weak infrastructure and political instability. Future projects should incorporate systematic approaches to cultural adaptation, such as those proposed in Bernal et al.'s framework,[31] to ensure interventions remain both culturally appropriate and evidence-based. Scaling-up initiatives should embed a systems-thinking approach into their design and implementation from the outset, recognizing that enablers and barriers are interdependent. For example, adaptability was closely tied to financing structures, stakeholder engagement influenced both governance and service delivery, and digital innovations depended on workforce capacity and infrastructure. Addressing these dynamics in a coordinated way is essential to strengthen long-term sustainability."

Comment 20. Line 395 – Can you please explain further how the systems thinking approach can be applied and how that relates to your work?
[Response] Thank you for raising this point. We have clarified our use of systems thinking in to ensure clarity for the reader. In the introduction we now explain that systems thinking in our study refers to recognizing the interdependencies between enablers and barriers across different domains of implementation and health systems, rather than treating these as isolated factors. We explain this in lines 115–122: "*Building on insights and gaps identified in the preliminary research,[18,21] this follow-up study extends earlier findings on enablers and barriers to scaling up NCD interventions. The first study applied systems thinking to identify challenges during implementation, while the second focused on prioritizing and planning for scale-up. Both contributed to the development of an extraction tool for systematically capturing factors influencing scale-up, including intervention characteristics, adaptation, stakeholder consultation, and sustainability. In the present study, we apply this tool within the CFIR 2.0 framework to examine projects that were nearing or completing scale-up, and systematically map barriers and enablers to the WHO health system building blocks."*
We also expand on this in the discussion in lines
In the discussion (lines 520–529)**,** we expand on this by illustrating how our findings demonstrated such interdependencies — for example, adaptability was shaped by financing structures, stakeholder engagement influenced both governance and service delivery, and digital innovations depended on workforce capacity and infrastructure. Together, these revisions make clear how a systems-thinking approach was applied in our analysis and how it strengthens the interpretation of our findings. Lines 520-529: *"Stakeholder burnout and the interplay between financing and system components, while recognized in prior research, were strongly reinforced by our findings, particularly in LMIC contexts where these challenges were compounded by weak infrastructure and political instability. Future projects should incorporate systematic approaches to cultural adaptation, such as those proposed in Bernal et al.'s framework,[31] to ensure interventions remain both culturally appropriate and evidence-based. Scaling-up initiatives should embed a systems-thinking approach into their design and implementation from the outset, recognizing that enablers and barriers are interdependent. For example, adaptability was closely tied to financing structures, stakeholder engagement influenced both governance and service delivery, and digital innovations depended on workforce capacity and infrastructure. Addressing these dynamics in a coordinated way is essential to strengthen long-term sustainability*."

Comment 21. Line 454 – I think I understand what you mean, but I think this section needs to be rephrased, as even when an intervention is designed to align with health system priorities, it can still encounter barriers, particularly from those that are outside the control of those running the intervention. For example, just because the intervention aligns with health system priorities, does not mean political instability wont still be a barrier.
[Response] Thank you. This is an important point. We agree that alignment with health system priorities is necessary but not sufficient to ensure successful scale-up. We have therefore revised this section to emphasize that alignment must be paired with strategies to mitigate these external barriers. The revised text can be found in lines 597-608: "*Aligning interventions with health system priorities such as service delivery, financing, and governance is essential for*

scale-up, but not sufficient on its own. Even well-aligned programs may encounter barriers beyond the control of implementers, including political instability, resource limitations, or fragmented systems, which can ultimately hinder their impact. Policymakers and practitioners should therefore prioritize health system strengthening as a foundation for scaling up NCD interventions, while recognizing that external factors may still pose challenges. In practice, early and sustained engagement of local stakeholders, including healthcare providers and community members, fosters ownership and supports long-term success. Strategies should also prevent stakeholder burnout, maintain engagement throughout the intervention lifecycle, and simplify intervention models to reduce complexity, particularly in decentralized systems. Finally, addressing financial constraints through sustainable financing mechanisms and collaboration between policymakers and implementation teams is critical for ensuring continuity and expansion."

Comment 22. Line 489 – This last line is the first mention of "co-design". Maybe worth rewording to more directly align with the rest of the paper rather than introducing a new concept.
[Response] Thank you for this observation. We agree that introducing "co-design" in the final line could be confusing, as it may appear as a new concept rather than a continuation of earlier findings. To improve clarity and consistency, we revised line 648 to the following: "..fostering community ownership *through sustained stakeholder engagement and cultural adaptation."*

Reviewer 2

We thank the second reviewer for the thoughtful review and constructive comments, which greatly helped clarify the contribution of this study.

Major concerns:
Comment 1. A danger of working with such a large database is that information becomes redundant without adding new insights. The authors appear to be aware of the danger and highlight which new insights the paper is adding. e.g. Building on these insights and gaps identified in the preliminary research,18,21 this follow-up study explores opportunities to advance previous findings. Will be beneficial for reader if the authors could contextualise the study. How are the three papers linked? That will the provide an opportunity to clearly define tools that were developed to extract and analyse data.
[Response] Thank you for this important comment. We agree that it is essential to contextualize how this study builds on previous publications from the GACD scale-up working group and to make clear what is novel in this manuscript. We therefore revised the Introduction to clarify the relationship between the three papers. Specifically, we explain that the first study (Ramani-Chander et al. 2022) applied systems thinking to identify barriers and enablers during implementation, while the second study (Ramani-Chander et al. 2023) focused on prioritizing and planning for scale-up. Both studies contributed to the development of an extraction tool that systematically captured factors influencing scale-up, such as intervention characteristics, adaptation, stakeholder consultation, and sustainability. In the present manuscript, we extend this work in three ways: (i) by applying the extraction tool within the CFIR 2.0 framework to examine projects that were nearing or completing scale-up, (ii) by systematically mapping barriers and enablers to the WHO health system building blocks, and (iii) by generating new insights into sustainability and long-term embedding of interventions. This clarification highlights the continuity between the three papers, while also demonstrating that the current study provides unique contributions beyond what has already been published. We have revised the text in the introduction in lines 115-130 to: "Building on insights and gaps identified in the preliminary research,[18,21-23] this follow-up study *extends earlier findings on enablers and barriers to scaling up NCD interventions. The first study applied systems thinking to identify challenges*

during implementation, while the second focused on prioritizing and planning for scale-up. Both contributed to the development of an extraction tool for systematically capturing factors influencing scale-up, including intervention characteristics, adaptation, stakeholder consultation, and sustainability. In the present study, we apply this tool within the CFIR 2.0 framework to examine projects that were nearing or completing scale-up, and systematically map barriers and enablers to the WHO health system building blocks. Although others have highlighted enablers and barriers to scaling up NCD interventions in LMICs, achieving sustainable and widespread implementation continues to pose significant challenges. *By focusing on health system factors critical for scalability and sustainability, this study provides new insights using a framework into long-term strategies for embedding and expanding NCD-related initiatives across diverse contexts.*"

Comment 2. It is not clear why you used the CFIR framework. Please motivate - did you find the WHO building block structure more beneficial? Please explain
[Response] We chose CFIR 2.0 as our primary framework because it provides a detailed, implementation science–based structure for identifying enablers and barriers across multiple domains, with an explicit focus on scalability and sustainability. The WHO building blocks, while widely used for health system analysis, are less granular for capturing implementation processes. To benefit from both perspectives, we used CFIR 2.0 for coding and analysis, and subsequently mapped results to the WHO building blocks to situate findings within a health systems context. We added the following text in lines 148-152: "*We applied CFIR 2.0 as our primary framework because it provides a comprehensive structure for identifying barriers and enablers with a focus on scalability and sustainability. The WHO building blocks[12] offer a systems-level perspective but are less detailed for implementation processes. To combine these strengths, we analyzed data using CFIR 2.0 and subsequently mapped findings to the WHO building blocks.*"

Comment 3. The limitation identified by authors regarding stakeholder identification is VERY important. Does not allow a true evaluation of the individual factors in CFIR. Supports question of which new insights are offered
[Response] We agree that the absence of input from certain key stakeholders, such as community members, nurses, and policymakers, restricted our ability to evaluate CFIR constructs fully at the individual level. This limitation reflects the recruitment process, which primarily relied on invitations through principal investigators, and may have biased the sample toward more successful projects. We have now made this explicit in the Limitation in lines 585-590. At the same time, we emphasized that the study still generates new insights by focusing on projects nearing or completing scale-up, integrating our previously developed extraction tool with CFIR 2.0, and mapping results to the WHO building blocks. These steps allowed us to provide novel contributions despite the limited individual-level stakeholder representation.
Lines 585-590: "…The absence of input from these critical voices may have limited the understanding of the real-world barriers and successes associated with the interventions. *It also constrained our ability to fully evaluate CFIR constructs at the individual level, as perspectives from key actors were missing. Nevertheless, by focusing on projects nearing or completing scale-up, integrating our extraction tool with CFIR 2.0, and mapping results to the WHO building blocks, we were still able to generate novel insights into systemic enablers and barriers influencing scalability and sustainability.*"

Minor concerns:

Comment 4. Some areas of the methodology also include results for example the documents submitted for analysis. Table 1: results

[Response]. Table 1 could be interpreted as presenting results. However, our intention is to provide descriptive context for the dataset analyzed, rather than to report analytical findings. To make this clearer, we added text to explicitly state that Table 1 summarizes the characteristics of the projects and documents included in the study dataset, thereby distinguishing it from the analytical findings reported in the Results. The added text in lines 185-187 follow: "*Table 1 provides a descriptive overview of the projects and documents included in the dataset (e.g., WHO region, intervention type, number of documents). These characteristics are presented here to contextualize the dataset."*

Comment 5. It is not clear if interviews were linked to document review of the specific project? E.g "The interviews focused on underreported or unclear information from documentation related..."

[Response] We have clarified in the methods that interviews were directly linked to the document review of the same projects. Specifically, interviews were used to complement and clarify the documentary data, focusing on areas where documents were limited or unclear (e.g., contextual adaptations, sustainability planning, stakeholder engagement). This ensured that data collection across sources was integrated and project-specific rather than separate streams. We have clarified this in lines 180-184: "*Data collection involved a combination of document review and semi-structured interviews for each project. Interviews were conducted with project representatives to complement the documentary data, focusing on information that was underreported, unclear, or missing in the documents (e.g., stakeholder perspectives, contextual adaptations, sustainability planning). This linkage ensured that the interview and document data were integrated and project-specific."*

Comment 6. Authors refer to different systems involved in sustainability but then only refer to "health care systems". Which other systems did authors identify?

[Response] Thank you for pointing this out. We agree that our wording implied only "health care systems", whereas our findings also highlighted the role of broader systems beyond health, particularly social systems, community structures, and policy/governance systems that intersect with health interventions. We have therefore added the following text in lines 488-492: "*While healthcare systems were central to the sustainability of interventions, our findings also pointed to the importance of broader systems, including social and community structures, education systems, and policy/governance mechanisms. These intersecting systems played a critical role in enabling scale-up and sustainability, for example by mobilizing communities, embedding interventions in trusted local networks, and aligning health initiatives with broader social development priorities*."

Comment 7. Not sure on which data the following strong statement is based: "Scaling-up initiatives must embed a systems-thinking approach into their design"

[Response]. We have revised this section of the to ensure that the statement is directly grounded in our findings and not presented as an unsupported prescription. The text now emphasizes that our results reinforce the value of a systems-thinking approach, highlighting the interdependencies observed across CFIR domains and WHO building blocks. For example, adaptability was tied to financing structures, stakeholder engagement influenced both governance and service delivery, and digital innovations depended on workforce capacity and

infrastructure readiness. By illustrating these interconnections, the revised text makes clear that our data support systems thinking as an important interpretive lens for scale-up. The revised text can be found in lines 525-529: "*Scaling-up initiatives should embed a systems-thinking approach into their design and implementation from the outset, recognizing that enablers and barriers are interdependent. For example, adaptability was closely tied to financing structures, stakeholder engagement influenced both governance and service delivery, and digital innovations depended on workforce capacity and infrastructure. Addressing these dynamics in a coordinated way is essential to strengthen long-term sustainability.*"

Comment 8. Please differentiate between "co-design" of intervention and "contextualize" intervention. Which concept emerged from your data?
[Response] We agree that our original wording implied that co-design was a central theme, which could be misleading. Our findings primarily reflected contextualization as a consistent enabler of scalability and sustainability. While a few projects included participatory features that resembled co-design, these were less common, and the dominant pattern was continuous adaptation to context as interventions expanded. To clarify this, we revised the following text in lines 453-458: "*For example, our findings highlight the importance of adaptability and the ongoing contextualization of interventions to address cultural misalignment, a nuance not fully explored in earlier studies. While a few projects included participatory elements that resembled co-design, the dominant pattern was the adaptation of interventions to local contexts as they scaled. Additionally, the study sheds light on innovative approaches such as leveraging digital health tools to sustain interventions during disruptions, like those caused by the COVID-19 pandemic.*"

Comment 9. I was unable to locate the paper: Agyepong IA, Adjei S. Addressing the NCD burden in LMICs: The role of the Global Alliance for Chronic Diseases. Health Policy Plan. 2022;37(2):160–7. Please check also all references.

[Response] Thank you for this comment. We confirmed that the original reference was incorrect, as no such publication exists. We have removed it and replaced it with an appropriate alternative:

> Aziz, Z., Colquhoun, H., McCutchan, G., et al. (2022). The Global Alliance for Chronic Diseases Implementation Science School: building capacity in LMICs. International Journal of Public Health, 67:1604944. https://doi.org/10.3389/ijph.2022.1604944

This paper highlights GACD's role in strengthening implementation science capacity in LMICs, which is consistent with the point we intended to support. In addition, we re-checked all references in the manuscript to ensure accuracy and verifiability.

VERSION 2 - REVIEW

**Reviewer**	**2**
**Name**	**Hanekom, Susan**
**Affiliation**	**Stellenbosch University, Department of Physiotherapy**
**Date**	**06-Oct-2025**

COI

Thank you for addressing concerns
